# Arabidopsis Flower and Embryo Developmental Genes are Repressed in Seedlings by Different Combinations of Polycomb Group Proteins in Association with Distinct Sets of Cis-regulatory Elements

**DOI:** 10.1371/journal.pgen.1005771

**Published:** 2016-01-13

**Authors:** Hua Wang, Chunmei Liu, Jingfei Cheng, Jian Liu, Lei Zhang, Chongsheng He, Wen-Hui Shen, Hong Jin, Lin Xu, Yijing Zhang

**Affiliations:** 1 National Laboratory of Plant Molecular Genetics, CAS Center for Excellence in Molecular Plant Sciences, Institute of Plant Physiology and Ecology, Shanghai Institutes for Biological Sciences, Chinese Academy of Sciences, Shanghai, China; 2 Department of Chemistry, Fudan University, Shanghai, China; 3 State Key Laboratory of Genetic Engineering, Collaborative Innovation Center of Genetics and Development, International Associated Laboratory of CNRS-Fudan-HUNAU on Plant Epigenome Research, Department of Biochemistry, Institute of Plant Biology, School of Life Sciences, Fudan University, Shanghai, China; 4 Institut de Biologie Moléculaire des Plantes, UPR2357 CNRS, Université de Strasbourg, Strasbourg, France; 5 Institute of Biomedical Science, Fudan University, Shanghai, China; IBENS, FRANCE

## Abstract

Polycomb repressive complexes (PRCs) play crucial roles in transcriptional repression and developmental regulation in both plants and animals. In plants, depletion of different members of PRCs causes both overlapping and unique phenotypic defects. However, the underlying molecular mechanism determining the target specificity and functional diversity is not sufficiently characterized. Here, we quantitatively compared changes of tri-methylation at H3K27 in Arabidopsis mutants deprived of various key PRC components. We show that CURLY LEAF (CLF), a major catalytic subunit of PRC2, coordinates with different members of PRC1 in suppression of distinct plant developmental programs. We found that expression of flower development genes is repressed in seedlings preferentially *via* non-redundant role of CLF, which specifically associated with LIKE HETEROCHROMATIN PROTEIN1 (LHP1). In contrast, expression of embryo development genes is repressed by PRC1-catalytic core subunits AtBMI1 and AtRING1 in common with PRC2-catalytic enzymes CLF or SWINGER (SWN). This context-dependent role of CLF corresponds well with the change in H3K27me3 profiles, and is remarkably associated with differential co-occupancy of binding motifs of transcription factors (TFs), including MADS box and ABA-related factors. We propose that different combinations of PRC members distinctively regulate different developmental programs, and their target specificity is modulated by specific TFs.

## Author Summary

Polycomb group proteins (PcGs) are essential for development in both animals and plants. Studies in plants are advantageous for elucidation of specific effects of PcGs during development, since most PcG mutants are viable in plants but not in animals. Previous efforts in genetic study of plant PcGs revealed that different PcGs have both common and unique effects on plant development, but the mechanisms underlying the specific regulation of different developmental programs by PcGs are still far from clear. In this study, we quantitatively compared the change in H3K27me3 and gene expression profiles between mutants of key PcG members on a genome-wide scale in Arabidopsis seedlings, and successfully unraveled different developmental programs that are specifically regulated by different combinations of PcGs. This context specific effect of PcGs is closely associated with different sets of transcription factor binding motifs. Together, we revealed on a genome-wide scale that different combinations of PcGs, as well as their association with the binding sites of different TFs, serve to explain the specific regulation of different developmental programs by PcGs.

## Introduction

The evolutionarily conserved Polycomb group proteins (PcGs) are the major epigenetic machinery regulating differentiation and development [1–4]. PcGs mediated repression is achieved by establishment and maintenance of epigenetic modifications surrounding target genes. In both plants and animals, PcGs are classified into two major multi-protein complexes PRC1 and PRC2, which participate in transcriptional repression by catalyzing H3K27 tri-methylation and H2A ubiquitination, respectively [1–4]. Depletion of various PcG components in plants lead to varied developmental defects [5–13]raising a major question about how the functional specificity of PcGs is established.

Study of the functional specificity of PcGs in both plant and animals is non-trivial. Firstly, the majority of PcG components are ubiquitously expressed, and do not have sequence-specific DNA recognition properties. Secondly, members of PcGs generally have functional redundancy and diversity, and it is difficult to distinguish the specific effect of individual members. Plants are advantageous for studying the effect of PcGs in development since most plant PcG mutants are viable, while animal development is generally vulnerable to PcG mutations. Previous efforts in genetic dissection of PcGs’ functions provide important clues as to the specialized functions of PcG members. For example, CURLY LEAF (CLF) and SWINGER (SWN) are two highly similar enzymatic subunits of the PRC2 complex [11], and they play redundant roles in plant development as double mutants *clf swn* show a much more severe phenotype than each of the single mutants [11]. However, this redundancy is partial since SWN cannot rescue the phenotypic defects upon loss of *CLF*, including early flowering and curly leaf [11]. Similarly, lack of either *AtRING1* or *AtBMI1*, the core catalytic factors of PRC1, leads to depression of embryonic traits in seedlings, while only the *atring1a atring1b* double mutant displays severely fused flower phenotype [12,13]. In addition, LIKE HETEROCHROMATIN PROTEIN1 (LHP1, also known as Terminal Flower-2, TFL2) is a PRC1 component [10,14,15] capable of interacting with both PRC1 components AtBMI1 and AtRING1 in vitro [12,13,16]. However, *lhp1* displays some similar phenotypic defects to those of the PRC2 mutant *clf*[10], and more recent evidence showed that LHP1 co-purifies with PRC2 complex [17]. Moreover, studies on different loci led to different conclusions on the interplay between PRC1 and PRC2, including their orders of recruitment into the corresponding complexes [7,18,19]. It seems that different PcGs tend to repress specific gene sets with distinct functions. However, target genes and the mechanisms of specificity for different PcGs are not sufficiently characterized, particularly from a genome-wide point of view.

Multiple mechanisms had been proposed in Arabidopsis to explain the target specificity of PcGs, *e*.*g*. specific recruitment of PcGs by diverse strategies for transcription repression [7,20–27] and selective displacement of PcGs by some transcription factors for transcription de-repression during developmental transitions [28,29]. However, most of these proposed mechanisms are based on studies of some specific genes without an overview at genome-wide scale. It is also worth to note that some conclusions drawn from genome-wide studies may not be always consistent with results obtained at specific gene loci. An example case is regarding the H3K27me3 modification change in the *lhp1* mutant. The global H3K27me3 pattern in the mutant was similar to that in wild-type Col-0 plant, leading to a conclusion that LHP1 is responsible for recognizing H3K27me3 and facilitating PRC1 binding but not for depositing H3K27me3 [14]. However, some more recent ChIP-qPCR results revealed that several PRC2 targets show an obvious reduction of H3K27me3 levels in *lhp1*[17,23]. This discrepancy between genome-wide and ChIP-qPCR results could be due to H3K27me3 differences in *lhp1* being localized to some specific genomic regions, which had been missed in detection by genome-wide profiling with relatively low resolution. Therefore, for unraveling the locus selectivity and distinguishing the specific effects of different PcGs, combining high-resolution genome-scale data with quantitative analyses methods are indispensable.

The next-generation sequencing technology has enabled the detection of epi-genome profiles with high sensitivity and specificity [30–32]. We have recently developed a package for quantitative comparison of epi-genomic data, showing a high quality in dissecting specific epigenomic modifications in animal development [33–36]. Using these newly developed methods, here we quantitatively compared genome-wide changes of H3K27me3 and gene expression profiles in loss-of-function mutants in PRC1 (AtBMI1, AtRING1 and LHP1) and PRC2 (CLF) components in *Arabidopsis* seedlings. We revealed that CLF collaborates with different PRC1 subunits to repress flower and embryo development. We further demonstrated that the target specificity of these different combinations of PcGs is closely associated with different sets of TF binding motifs, pointing to an active interplay between particular TFs and the specific activity of different PcGs.

## Results

### LHP1 co-purified with FIE PRC2 complex

To dissect the composition of PRC2 complex in Arabidopsis, we used the FIE-3XFLAG fusion protein in immunoprecipitation experiments to identify associated proteins from leaf explants cultured in callus-induction medium. Following mass spectrometry analysis (see Methods), we identified the well-documented PRC2 components, including SWN, CLF, EMF2 and VRN2, as well as the previously considered PRC1 component LHP1 (S1 Table), consistent with recent report in inflorescence [17], indicating that the interaction between LHP1 and PRC2 complex is relatively stable across different tissues. It is worth noting that although LHP1 has the ability to bind the core catalytic subunits of PRC1 including AtBMI1 and AtRING1 *in vitro*[12,13,16], neither component was identified here, suggesting a special role for LHP1 in association with PRC2 complex.

### Different PcG subunits have non-redundant roles in repressing particular gene sets via H3K27me3

**Fig 1:**
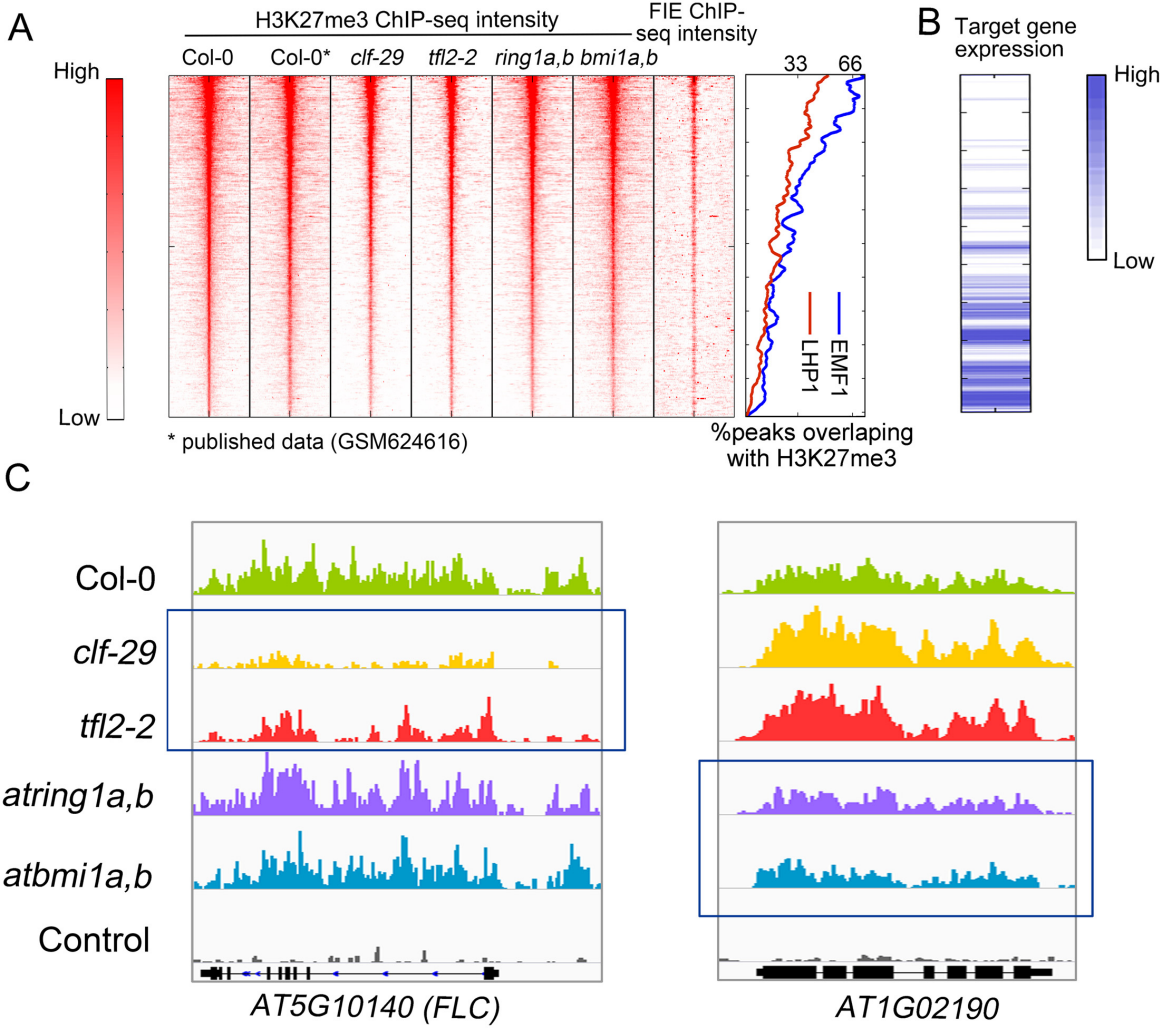
Local difference of H3K27me3 marks in mutants of PcG components. (A) ChIP-seq density heatmaps in Col-0 and PcG mutants, ranked by H3K27me3 read intensity within ±500 bp of peak summits in Col-0. The Pearson correlation coefficient between H3K27me3 intensity in Col-0 and FIE binding within ±50 bp of peak summits is 0.52. ChIP-seq data of H3K27me3 in panel II and FIE binding in panel VII were published previously [40,41]. To compare H3K27me3 level with the bindings of LHP1 and EMF1 based on published ChIP-chip data [15,32], the fraction of EMF1 peaks (blue) or LHP1 peaks (red) Col-0 overlapped with Col-0 H3K27me3 peaks were plotted. (B) RNA-seq intensity heatmap for the targets of H3K27me3 peaks in Col-0 with the same order as in (A). The expression intensity is measured by Reads Per Kilobase per Million of mapped reads (RPKM). (C) (C) IGV screen shots showing the distribution of H3K27me3 mark intensity in Col-0 and different mutants of PcG components. Negative control is Col-0 sample immunoprecipitated with beads but without antibody. Regions showing quantitative reduction of H3K27me3 marks are highlighted by blue box.

To compare roles of different PcGs, we used chromatin immunoprecipitation followed by high-throughput sequencing (ChIP-seq) to characterize the genome-wide profiles of H3K27me3 in Col-0, *clf-29*, *tfl2-2*, *atbmi1a*,*b*, and *atring1a*,*b* (S2A Table). In Col-0, 5,055 read enriched H3K27me3 regions (peaks) were identified (S2B Table), 84% of which localized in promoter and genic regions (S1 Fig). The top enriched functions for those peak targets include transcription regulation, carpel and petal development (S2C Table). Notably, almost half of the annotated MADS box proteins are marked by H3K27me3 in seedlings (S2C Table). To compare H3K27me3 marks across samples, we first ranked the read intensities of Col-0 H3K27me3 peaks from high to low (Fig 1A panel I), which is positively correlated with the binding of PcG components including FIE, EMF1 and LHP1 (Fig 1A panel VII), and is inversely associated with the expression level of surrounding genes (Fig 1B). Next, the level of H3K27me3 mark in the corresponding regions in mutants were plotted side-by-side with that of Col-0 (Fig 1A panel III-VI), which showed highly similar patterns, with the global level slightly lower in some mutants as compared to Col-0. Similarly, slight reduction of H3K27me3 has also been reported in Arabidopsis mutant lacking *EMBRYONIC FLOWER*1 (*EMF1*)[32], a plant specific PcG member [37]. These results indicate that lack of any of these PcGs does not lead to complete loss of H3K27me3.

**Fig 2:**
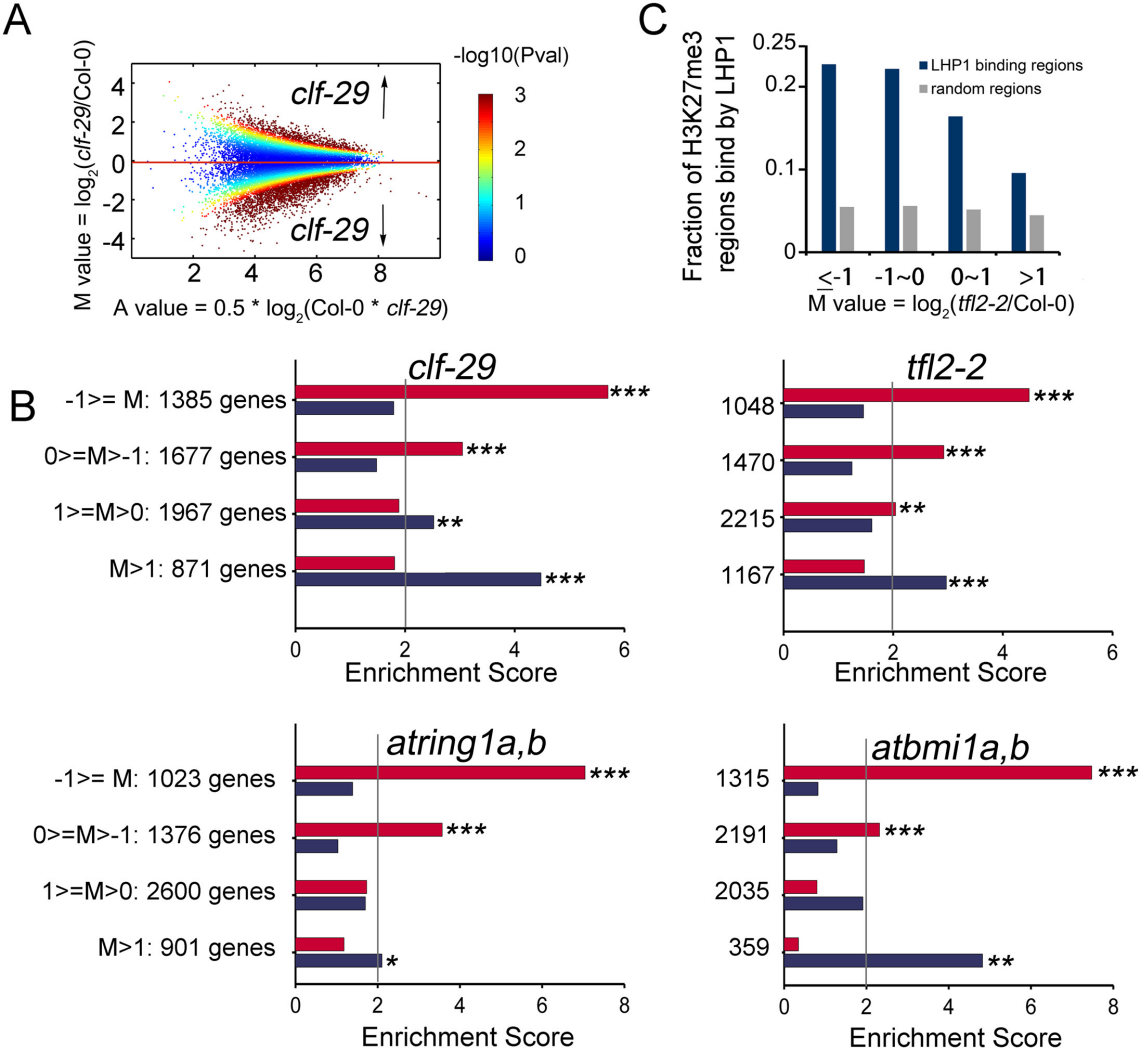
Quantitative difference of H3K27me3 marks are inversely correlated with the change of target gene expression in PcG mutants. (A) MA plot of all peaks from comparison of *clf-29* and Col-0 after normalization by MAnorm. Each dot represents a peak. X-axis is the A value, which represents the average intensity. Y-axis is the M value, which represents the difference of the intensity. Here, positive M value indicates higher H3K27me3 level in *clf-29* as compared to that in Col-0, and negative M value represents lower H3K27me3 level in *clf-29*. The color range represents -log10 P value associated with normalized peaks. (B) Enrichment of H3K27me3 peak targets with different M values in gene sets whose expression are regulated by different PcGs. The target genes were grouped by the M values of nearby peaks. For each group, the overlap with differentially expressed genes in a given PcG mutant was compared to the expected overlap at random; x-axis represents enrichment score. Fishers’ exact test was used to test the significance of overlap. *, P value <5e-2; **, P value <1e-2; ***, P value <1e-3. (C) Bar plot showing the percentage of H3K27me3 peaks with different M values in *tlf2-2* overlapped with LHP1 binding sites.

However, we observed different sets of loci showing apparent reduction of H3K27me3 in Arabidopsis deprived of different PcG members, suggesting PcG subunits have non-redundant roles in H3K27me3 deposition in local regions (Fig 1C). Traditional peak overlap method showed poor performance in characterizing H3K27me3 changes in PcG mutants (S2 Fig). Thus, we used the pipeline of MAnorm, a software package specifically designed for quantitative comparison of ChIP-seq datasets [33]. MAnorm derives its power from definition of M value, a statistic characterizing the strength of differential binding, the higher the absolute M value, the larger the difference, with the sign (+/-) representing higher intensity in PcG mutants or Col-0. In *clf-29*, we identified substantially more regions with reduced H3K27me3 modification than with increased H3K27me3 marks (Fig 2A), consistent with a major role for CLF in catalyzing H3K27me3. Intriguingly, similar pattern was also observed in *tfl2-2* and *atbmi1a*,*b* (S3 Fig), thus, PRC1 factors possibly contribute to H3K27me3 establishment. We also found hundreds of regions with increased H3K27me3 in *clf-29*, majority (68%) of which overlapped with H3K27me3 peaks in Col-0, while those non-overlapping regions also tend to be marked by H3K27me3 in Col-0, which are below peak detection cutoff, as shown by the read intensity distribution plot (S4 Fig). This increase of H3K27me3 in *clf-29* could be possibly due to the effect of other PcGs. Previous studies in both human and *Drosophila* also observed hyper-methylation of H3K27 in Ezh2 mutants [38,39], but no consensus has been made about the underlying mechanism.

To test if the quantitative difference of H3K27me3 has an effect on differential expression of target genes, H3K27me3 regions were partitioned to consecutive groups ranked by M value, and the gene targets for each group were identified (see Methods). The percentage of genes showing differential expression, up or down regulation separately, are depicted for each group (Fig 2B). In general, target genes associated with negative M value—that is, peaks with reduced H3K27me3 in mutants—were enriched in genes more highly expressed in mutants, and vice versa, which is consistent with the repressive role of H3K27me3. This indicates that the M value determined by MAnorm reflects authentic H3K27me3 changes.

Since loss of *LHP1* affects the H3K27me3 level for thousands of genes, we wondered whether the effect of LHP1 on H3K27me3 is direct or indirect. Previous evidence suggested that LHP1 co-localizes with H3K27me3 [14,15]. We compared the overlap between LHP1 binding peaks and genomic regions with change of H3K27me3 in *tfl2-2* characterized by M value (Fig 2C). Notably, nearly 1/4 regions with reduced H3K27me3 level (M<0) overlap with LHP1 binding sites as determined by previous ChIP-chip study [15]. The overlap is significantly higher (P value < 1e-3) than expected by chance based on permutation test (see Methods). In addition, we observed that the higher the effect of LHP1 on deposition of H3K27me3—as indicated by low M values—the higher the percentage of corresponding H3K27me3 regions overlapping with LHP1 binding, indicating a direct relationship between LHP1 binding and H3K27me3 change.

### Different sets of H3K27me3 loci are preferentially regulated by distinct combinations of PcGs

**Fig 3:**
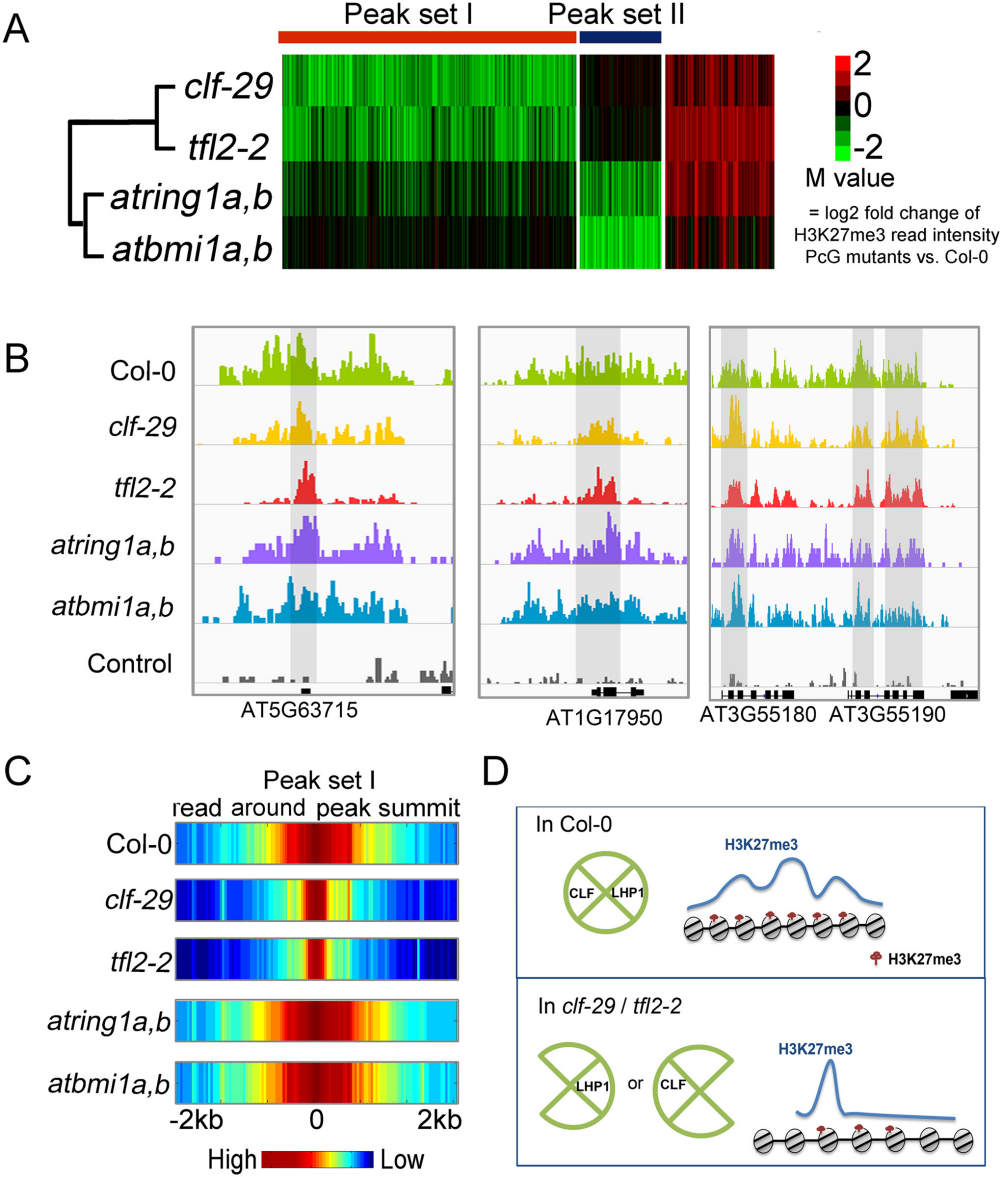
*clf-29* and *tfl2-2* show coordinated H3K27me3 change profiles distinct from those in *atring1a*,*b* and *atbmi1a*,*b*. K-means clustering of M values characterizing the quantitative change of H3K27me3 in PcG mutants. Definition of H3K27me3 quantitative change regions were based on combined criteria |M|>1 and P value <1e-3. Green and red colors represent lower and higher H3K27me3 levels in PcG mutants compared to that in Col-0. IGV screenshots support CLF and LHP1 participate in H3K27me3 elongation. Grey areas represent regions where significant H3K27me3 reductions happen mainly in surrounding regions but not in summit regions in *tfl2-2* and *clf-29* compared to Col-0. It’s worth noting that no smooth should be applied while preparing data for IGV screenshots, or else the difference between Col and *clf-29* or *tfl-2* would not be as obvious as the raw data. (C) Visualization of the profile of average read intensity around peak summits. All regions from peak set I were aligned such that peak summit is in the center of each region. Next, average read intensities were calculated and plotted for each consecutive 50 bp. (D) The diagram illustrates the finding based on H3K27me3 ChIP-seq data comparisons that LHP1 and CLF participate in elongation of H3K27me3 mark.

To dissect the cooperation of these PcGs on H3K27me3 modification, we collected 3,289 H3K27me3 regions regulated by at least one PcG component, and clustered the M values of H3K27me3 change in these regions to 3 clusters (Fig 3A and S3A Table). Although LHP1 physically interacts with AtRING1 and AtBMI1 *in vitro*[12,13,16], the H3K27me3 change profile in *tfl2-2* is closely correlated with that of *clf-29*, with 1,982 regions (peak set I) showing concerted reduction of H3K27me3 in both mutants. On the other hand, loss of AtBMI1A and AtBMI1B or AtRING1A and AtRING1B specifically reduced H3K27me3 level in 566 regions (peak set II), which represents PRC2 target sites affected by AtBMI1 and AtRING1 directly or indirectly, but is independent of LHP1. Thus, the dependence of H3K27me3 on LHP1 seems tightly correlated with the specific effect of CLF. In addition, 741 regions belonging to class III show increased H3K27me3 levels.

It is remarkable to find that CLF and LHP1 have a coordinated effect on H3K27me3 modifications at many genome regions. We wondered how these two factors control H3K27me3 around similar loci. A closer look at regions in peak set I indicates that loss of *CLF* or *LHP1* lead to apparent reduction of H3K27me3 in surrounding regions, but for most cases the signal at the summit is only slightly reduced (Fig 3B). A similar finding was reported supporting CLF-dependent disperse of H3K27me3 around transgenes carrying AG regulatory sequences [42]. Heatmap in Fig 3C showed the average read intensity around summits of peak set I (Fig 3D). Statistical analysis detected this phenomenon for 51% and 53% H3K27me3 reduction regions in *clf-29* and *tfl2-2*, respectively (S3B and S3C Table), indicating that CLF and LHP1 participate in H3K27me3 spreading.

### The expression of genes involved in different developmental processes are regulated by distinct combinations of PcGs

**Fig 4:**
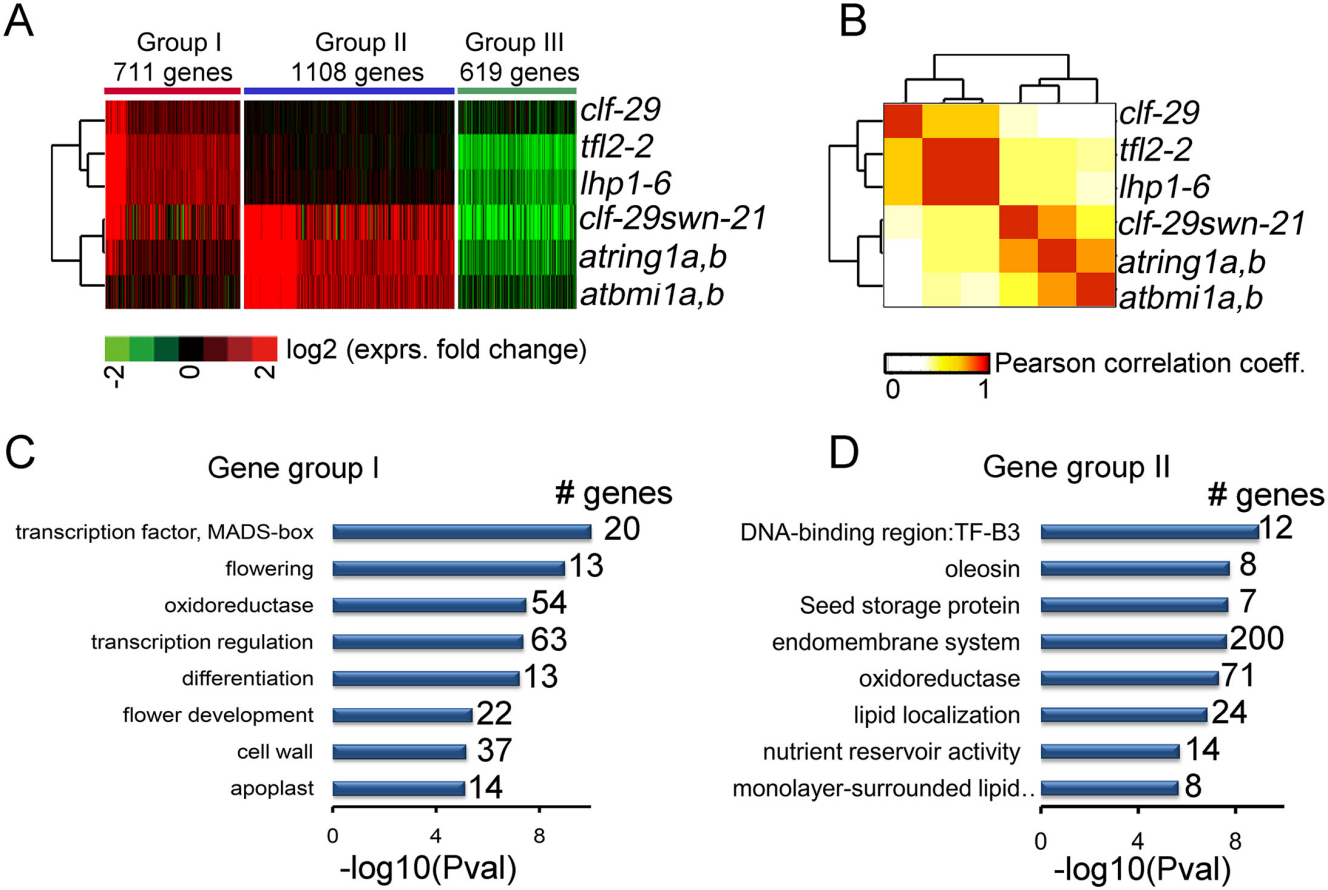
The profile of transcriptome change reveals two distinct combinations of PcGs with different functions. Three groups of genes showing distinct expression change profiles across mutants of PcG components. Green and red colors represent lower and higher expression in PcG mutants. Heatmap including another 1,290 genes whose expression affected only in *clf-29swn-21* is shown in S5A Fig. RNA-seq data sets were clustered via unsupervised hierarchical clustering based on Pearson correlation coefficients of log_2_ expression fold-change across samples. (C-D) Functional terms enriched in genes from group 1 (C) and group 2 (D) as shown in (A).

To investigate the functional consequence of the distinct H3K27me3 profile controlled by different combinations of PcGs, we first characterized the transcriptome change in each PcG mutant, including *atring1a*,*b*, *atbmi1a*,*b*, *clf-29*, *lhp1-6*,and *tfl2-2*. Next, 2,438 genes with differential expression in at least one of the five mutants were collected, and partitioned to 3 groups according to the expression change pattern across the 5 samples via k-means clustering (Fig 4A and S4A Table). Genes in group I are specifically up-regulated in *lhp1-6*, *tfl2-2* and *clf-29*, some of which are also induced in *atring1a*,*b* to some extent, but have no obvious change in at*bmi1a*,*b*. Comparison with expression change in *clf-29* and *swn-21* indicated that genes specifically increased in *clf-29* as compared to *swn-21* showing significant enrichment in group I (S5 Fig). Group II represents genes specifically higher expressed in *atring1a*,*b* and *atbmi1a*,*b*. Group III are genes repressed in all samples, which is perhaps not a direct effect of PcG components. It is interesting that genes from both group I and group II are upregulated in *clf-29swn-21* double mutants, whose transcriptome change is closely correlated with that in *atring1a*,*b* (Fig 4B), indicating RING1 and BMI1 regulated genes tend to be concertedly controlled by PRC2.

Next, we wondered whether genes from different groups participate in distinct functions or pathways. GO enrichment analysis showed that gene set of group I is closely related with flowering, floral development and transcription (Fig 4C), whereas genes in group II are involved in nutrient reservoir, seed storage, and lipid localization (Fig 4D). Enriched terms and genes are listed in S4B and S4C Table. These functional transcriptome analyses could serve to explain the specific phenotypic defects observed in corresponding PcG mutants.

### Different combinations of PcGs repress specific developmental programs via H3K27me3 remodeling of tissue-biased genes

**Fig 5:**
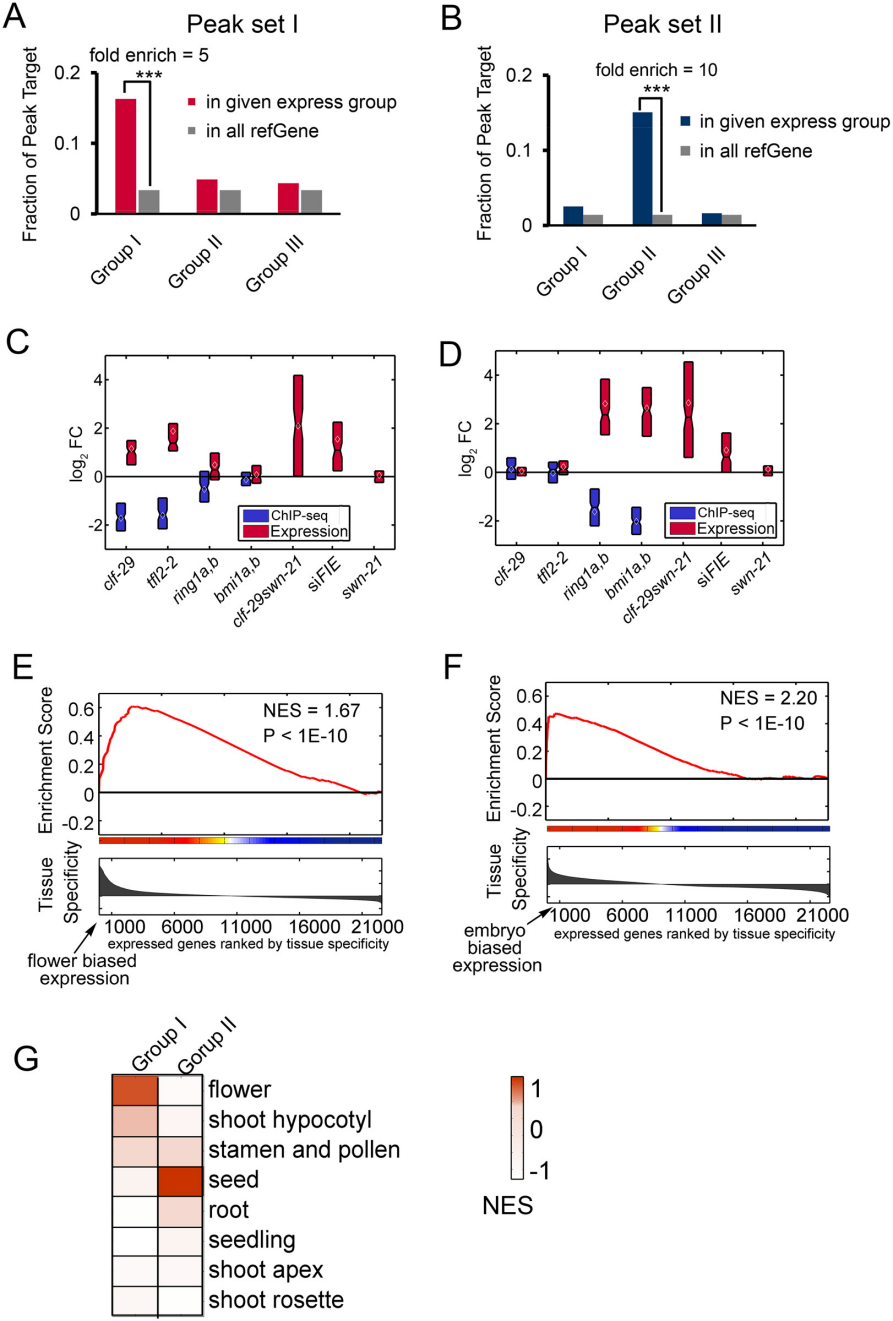
Targets of different combinations of PcGs and their expression bias. (A) Enrichment analysis of peak set I targets in three expression groups shown in Fig 4A. ***, Fishers’ exact test P value < 1e-3. (B) Enrichment analysis of peak set II targets in three expression groups shown in Fig 4A. ***, Fishers’ exact test P value < 1e-3. (C-D) Box plot showing the distribution of expression changes and H3K27me3 changes for 108 genes (C) and 164 genes (D) in PcG mutants. (E-F) Gene Set Enrichment Analysis (GSEA) showing a significant normalized enrichment score (NES) for flower biased expression of 108 genes (E) and embryo biased expression of 164 genes (F). The x axis represents all expressed genes ranked by tissue specificity as determined by expression profile, y axis presents the running enrichment score. (G) Heatmap showing NES calculated by GSEA for 108 genes and 164 genes in different gene sets with distinct tissue biased expressions.

To dissect the relationship between genes with distinct change profiles of H3K27me3 and differential expression, the targets for both peak set I and peak set II shown in Fig 3A were identified followed by statistical testing of their enrichment in each of the three expression groups identified in Fig 4A. As expected, genes in expression group I are significantly over-represented in targets of peak set I, representing genes showing decreased H3K27me3 marks and increased expression level in mutants of *CLF* and *LHP1*, and thus most likely to be the direct targets of CLF and LHP1 (Fig 5A). Similarly, genes in expression group II are preferentially enriched in targets of peak set II, representing genes affected by AtBMI1 and AtRING1 via H3K27me3 (Fig 5B).

To dissect the repressive function of PcGs via H3K27me3, 108 genes with increased expression and reduced H3K27me3 marks in mutants of *CLF* and *LHP1* are extracted (S5A Table). 164 targets regulated by AtBMI1 and AtRING1 were identified in the same way (S5B Table). The distribution of both expression and H3K27me3 change in different PcG mutants were plotted for the 108 and 164 genes, respectively (Fig 5C and 5D). The induced expression of 164 genes in *clf-29swn-21* double mutant but in neither *clf-29* nor *swn-7* confirms that CLF and SWN could complement each other’s function for these genes. It’s also worth noting that mutation of *CLF* but not *SWN* is responsible for the induction of 108 genes.

To investigate the function of the 108 and 164 genes during development, we first classified all tissues with gene expression information into 8 groups based on their gene expression profiles (S6 Fig). Next, we calculated the enrichment of the 108 and 164 genes in the different tissue-biased genes using Gene Set Enrichment Analysis (GSEA)[43]. The 108 genes are significantly more highly expressed in flowers, while the 164 genes are preferentially expressed in embryo (S7 Fig). GSEA using RNA-seq data showed consistent profiles with much lower P values (Fig 5E and 5F and S6 Table). Notably, neither gene sets was significantly enriched in other tissues (Fig 5G and S4 Fig), indicating a prominent relationship between PcGs and specific regulation of reproductive and embryo development.

### Context-dependent regulation of H3K27me3 by different PcG combinations is closely associated with binding motifs of specific TFs

We asked whether this different combination of PcGs is associated with different TFs. We started by searching for the enriched motifs surrounding all Col-0 H3K27me3 peak summits. The top enriched motifs include the binding motifs of ABI3 type transcription factor B3 (ABI3/FUS3/LEC1), ABI4, ABF1, SPL and MYB (S8 Fig). The first three are binding motifs of ABA related TFs, which mainly participate in embryo development [44–47], and is consistent with the well-documented function of PcGs in regulation of embryo development [12,16,31]. Despite the fact that some MADS-box TFs are reported to modulate specific H3K27me3 deposition for regulation of meristem identity, flowering and floral development in individual loci [20,22,23], CArG box, the binding motifs of MADS box transcription factors [48,49], show no enrichment when all H3K27me3 regions are considered (S2D Table).

**Fig 6:**
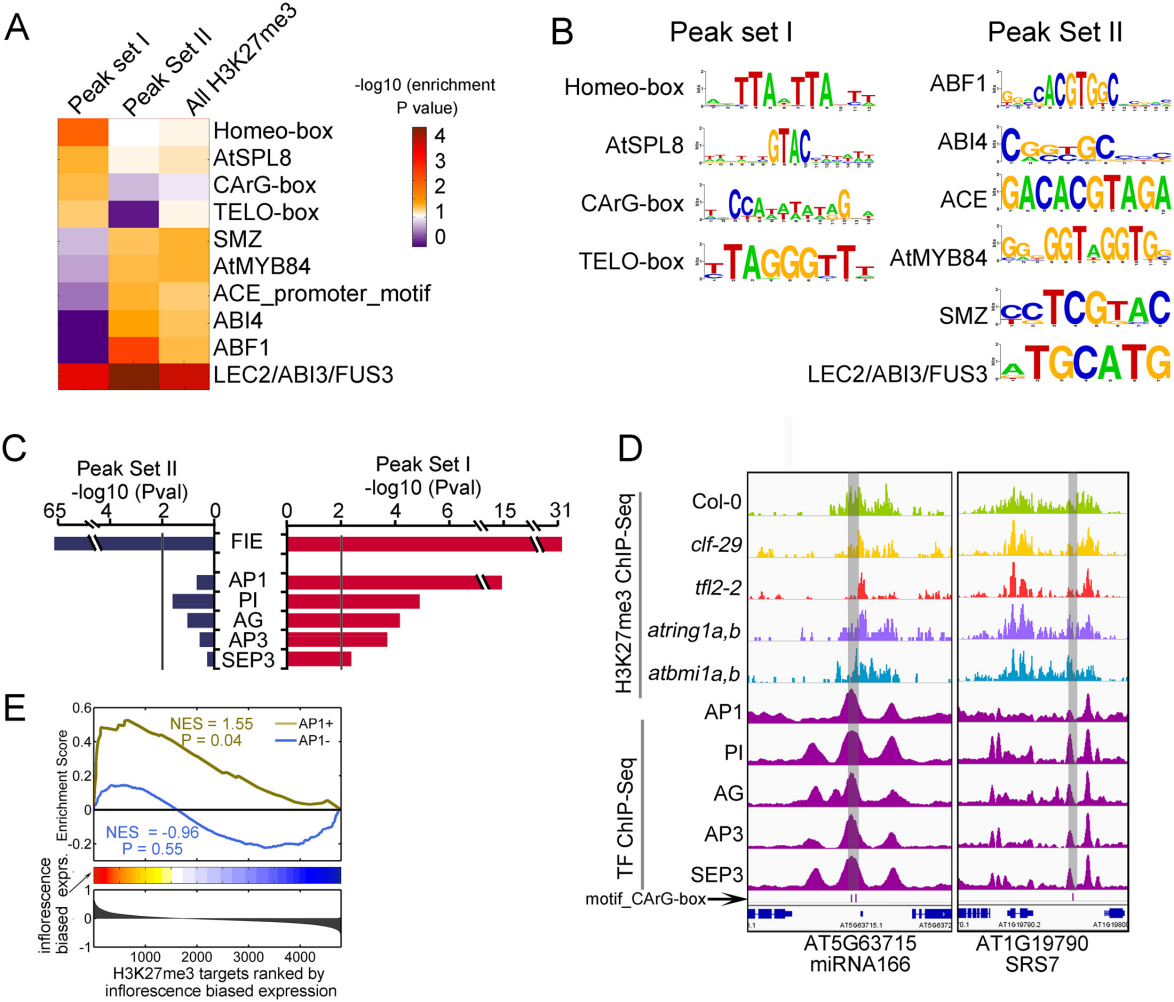
TF binding motifs and TFs whose bindings enriched in peak set I and peak set II. (A) Heatmap showing enriched motifs (P value < 0.01) in either peak set I or peak set II. Their enrichment in H3K27me3 peaks in Col-0 are also shown. (B) Sequence LOGOs of motifs enriched in peak set I and peak set II. (C) Enrichment of the binding sites of FIE and TFs in peak set I. (D) IGV screen shots showing examples of co-occupancy between H3K27me3 in seedlings and the bindings of MADS-box TFs in inflorescence. The motif sites of MADS box TFs are indicated by pink and blue bars at bottom of the screen shots, and are highlighted by grey area. (E) GSEA showing the common target genes of peak set I and AP1 binding peaks are preferentially expressed in inflorescence.

Next, we identified motifs over-represented in peak sets I and II (Fig 6A and 6B). Of note, binding motifs for MADS box and Homeobox were specifically enriched in peak set I, indicating a close relationship between transcription factors from these families and H3K27me3 levels synergically regulated by CLF and LHP1. On the other hand, motifs enriched in peak set II were similar to the result from all H3K27me3 peaks in Col-0, including ABI4 and ABF1 binding sites. The binding motif for B3 domain TFs are enriched in all H3K27me3 regions, to a higher extent in peak set II, consistent with the major role of their targets in embryogenesis, seed maturation and dormancy [50].

Since multiple TFs could bind to the same cis-regulatory sites, to identify TFs closely associated with different sets of peaks, we first collected and processed published ChIP-seq data characterizing TF binding profiles from Gene Expression Omnibus (GEO, http://www.ncbi.nlm.nih.gov/geo/). By querying against these processed binding sites, we found that the binding regions of some MADS box TFs are significantly enriched in peak set I but not in other H3K27me3 regions (Fig 6C and S7 Table). The top enriched TFs are floral organ identity genes, including AP1 [51], AG [52], AP3 [53] and SEP3 [51]. IGV screenshots in Fig 6D and S9A Fig illustrate some examples of co-occupancy between H3K27me3 in seedlings and these MADS-box TFs. The source of the TF ChIP-seq data, the enrichment statistics and co-occupied regions are listed in S7 Table. It should be noted that these TFs mainly expressed in inflorescence where these ChIP-seq data were generated from, while our ChIP-seq data were generated in seedlings. Thus, it is likely that specific binding of these TFs in some H3K27me3 regions from peak set I is responsible for selective de-repression of common target genes. In support of this, target genes of peak set I co-occupied by these TFs in inflorescence show apparent flower biased expression, while the other target genes of peak set I have no such expression bias (Fig 6E and S9B Fig), indicating the displacement of H3K27me3 by these TFs participating in activation of their common target genes. Consistently, it has been reported that some floral organ identity genes could interact with REF6 [54], the H3K27me3 demethylase in Arabidopsis [41], which possibly work together with MADS-box TFs to remove H3K27me3 marks. Taken together, our genome-wide analyses based on both motif and ChIP-seq data revealed that the bindings of MADS box TFs are closely associated with H3K27me3 peak set I regulated by CLF and LHP1, suggesting that it might be a widespread mechanism by which the specific activities of PcG family proteins is modulated by tissue specific TFs, resulting in distinct transcriptional outputs in different tissues.

## Discussion

**Fig 7:**
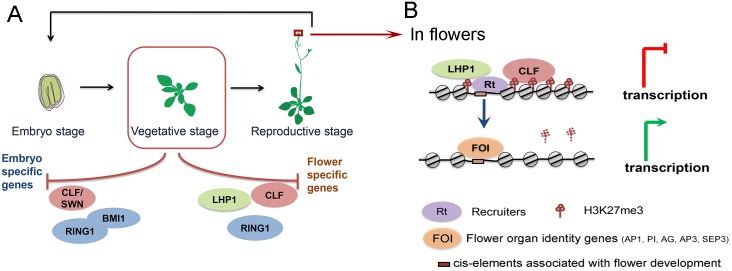
Working model for specific regulation of plant development by PcG components. (A) In seedlings, CLF and LHP1 work concertedly to repress flower specific genes, and some targets are also regulated by RING1. While repression of embryonic development requires cooperative regulation of AtBMI1, AtRING1, and the redundant role of CLF and SWN. (B) In inflorescence, significant parts of CLF and LHP1 target sites in seedlings are occupied by inflorescence specific TFs, which participate in de-repression of the common target genes.

In this study, based on quantitative comparison of epigenomic and transcriptomic data in mutants of core PcG components, we revealed that CLF collaborates with different PcGs partners to achieve transcriptional repression in distinct developmental programs. Importantly, target specificity of different combination of PcGs are closely associated with different sets of TF binding motifs, suggesting a widespread mechanism for modulation of PcGs specificity by particular TFs. We propose a context-dependent model for PcGs in selective repression of flower or embryo development (Fig 7).

### Distinct compositions of PcGs preferentially repress different transcriptional programs

As an ancient machinery for developmental regulation, PcGs employed multiple protein families which underwent duplication and diversification, and thus they share common targets but also have specialized functions. The common targets may represent those that are involved in more ancient processes in development, while the unique features may have evolved more recently. In support of this, we observed that embryo development, presenting in an overwhelming majority of land plant (embryophyte)[55], is regulated by majority core components of PRC1 and PRC2, while flower development, a relatively recently evolved process in plants [56], is specifically regulated by only a subset of PcGs, with LHP1 and CLF as the major players. Then does the emergence of LHP1 or CLF parallel the appearance of flowering plant (angiosperms)? LHP1 is present in ancient plant species including *Selaginella moellendorffii* and *Physcomitrella patens*[57]. Interestingly, our phylogenetic analysis revealed that the divergence of CLF and SWN likely occurred accompanying the emergence of angiosperms (S10 Fig), with the duplication existing in all angiosperm species collected, including *Amborella trichopoda*, the oldest known angiosperm, but not in more ancient species, including moss, *Selaginella* or plant from *Gymnospermae*. Thus, it is possible that after the duplication event, CLF preferentially acquired the ability of regulating floral development. Similarly, AtRING1 and AtBMI1, the catalytic subunits of PRC1, have both joint and individual functions. It is likely that in addition to the common targets with AtBMI1, AtRING1 also participate in regulating H3K27me3 modifications and expression for several target genes of CLF and LHP1 (Figs 3 and 4), which possibly serves to explain aberrant flower development only observed in *atring1a*,*b* but not *atbmi1a*,*b*[12,13].

Our finding that PRC2 composition regulates differential transcriptional programs is analogous to a recent genome-wide study about human blood cell development [34]. It was demonstrated that histone methyltransferase Enhancer of zeste1 (EZH1) and EZH2, the counterparts of CLF and SWN in human, form alternative PRC2 complexes with distinct subsets of PcGs, occupy different chromatin domains and regulate distinct transcriptional activities [34]. Given that epigenetic machineries generally have multiple family members, and thus could form a suite of different combinations, different compositions of epigenetic complexes could at least partially explain their selectivity in transcriptional regulation.

### Crosstalk between specific TFs and PcGs in developmental regulation

Target selection by PcGs in different developmental stages is critical for correct developmental regulation. Specific recruitment of PcGs by particular factors [7,20–23], Polycomb response elements (PREs)[26,27], or non-coding RNAs [24,25] have been a major explanation for both PRC1 and PRC2 binding in plants [58]. Recent reports proposed that release of PcG by a particular MADS protein AG is the prerequisite for activation of KNU [28,29]. Whereas all these conclusions are drawn from analyses based on limited loci, we provide clues from the genome-wide scale that MADS box TFs are possibly associated with specific release of PcGs. Specific recruitment of PcGs by MADS box TF has recently been reported. SHORT VEGETATIVE PHASE (SVP), the major flowering repressor in seedlings [59], was shown to be able to interact with LHP1, and contribute to *SEP3* repression via H3K27me3 [23]. Consistently, we found SVP binding sites [59] show good correlation with K27me3 modification in peak set I (S11 Fig). However, it’s binding sites are also enriched in peak set II, while the common binding sequences have no obvious relationship with CArG-box motifs. We observed that SVP ChIP-seq data have relatively high noise, and the binding regions tend to be broad, thus some coincident bindings could possibly be due to noise. Alternatively, SVP has diverse functions in addition to recruit PcGs for flowering repression.

MADS box TFs play a central role in flower development, and their family size increased explosively with the origin of angiosperm [60]. We found almost half of the Arabidopsis MADS box TFs have the H3K27me3 modification in seedlings, indicating a strict control of MADS gene expression by PcGs (S2C Table). On the other hand, we demonstrated that binding sites of MADS box TFs specifically enriched in genes controlled by CLF and LHP1, and possibly contribute to modulate the target selectivity. These findings close the loop of the transcription network regulating development processes, in which expression of tissue specific TFs is controlled by epigenetic marks, and specific TFs cooperate with epigenetic complexes in determining target selection and developmental regulation. How the epigenetic machinery co-evolved with specific TFs to cooperatively fine-tune the regulation of plant development is an intriguing phenomenon deserving further in-depth exploration.

It is intriguing that telo-box, a widespread short motif identical to the repeat (AAACCCT)_n_ of plant telomeres [61], is specifically enriched in peak set I (Fig 6A and 6B). Telo-box is reported to be involved in regulating gene expression in cycling cells [62], and whether the association between telo-box and the function of CLF or LHP1 involved in maintaining H3K27me3 marks during cell cycle is an interesting issue for further study. Genome-wide Hi-C analyses revealed telomeric regions and H3K27me3 modifications form local interactive hot spots [64]. And a recent study showed complementary activities of TELOMERE REPEAT BINDING proteins and PcGs in transcriptional regulation of target genes [63]. It is possible that telo-box also mediates local interaction among H3K27me3 marked regions. Collectively, our study provides rich resource as well as insightful clues for further exploration of the relationship between cis-elements, TFs and PcGs in specific regulation of developmental processes.

### Role of chromo-domain proteins in H3K27me3 spread and maintenance

The chromo-domain protein Heterochromatin protein 1 (HP1) and Polycomb (Pc) in animals bind H3K9me3 and H3K27me3, respectively [65–68]. Despite LHP1 sharing relatively higher level of sequence similarity with HP1, it can bind H3K27me3 in vitro and co-localize with H3K27me3 in vivo [14,15], and was originally proposed to be the counterpart of Pc, responsible for recruitment of PRC1 to H3K27me3 catalyzed by PRC2. If this is the case, LHP1 should function downstream of PRC2. However, recent studies showed that H3K27me3 modifications also require LHP1 [17,23]. Here, our genome-wide results revealed that H3K27me3 levels of thousands of loci are controlled by LHP1. Further quantitative comparison of H3K27me3 change profiles across PcG mutants revealed that the effects of LHP1 on H3K27me3 modification and target gene repression are coordinated with the non-redundant role of CLF (Figs 3 and 4). In addition, both CLF and LHP1 are involved in spread of H3K27me3 marks since loss of either component lead to localized H3K27me3 signals (Fig 3B, 3C and 3D). These findings not only confirmed previous report from studying AG transgene that CLF is indispensable for H3K27me3 spreading [42], but also identified LHP1 as an important cofactor with CLF in H3K27me3 elongation, which could finally contribute to inheritance and stability of epigenetic silencing.

Notably, LHP1 has no effect on genes jointly controlled by both PRC1 and PRC2 (targets of peak set II) despite the coincident binding of LHP1 and H3K27me3 in these regions. If the requirement of chromo-domain protein for H3K27me3 maintenance is a widespread mechanism, then there may be other chromo-domain proteins functional for spreading of H3K27me3 in these H3K27me3 regions. Alternatively, PcGs employ multiple strategies for H3K27me3 maintenance at different loci, either using LHP1 or cooperating with core subunits of PRC1 to create compacted chromatin structures [69]. There are 13 Arabidopsis proteins that have chromo-domain, and further epigenomic studies are required to have a deeper understanding about the role of chromo-domain proteins on epigenetic modifications. Due to the functional redundancy and localized effects of epigenetic machineries, the quantitative comparison pipeline applied in this study will be of great help for further exploration based on high throughput data.

## Methods

### Experimental procedures

#### Affinity purification of FIE complex

We constructed pFIE: FLAG-FIE transgenic plants (refer to S1 Text for details), and collected 5 g of leaf explants (8 days cultured in callus induced medium) from T2 plants. Next, FIE complex is purified using the FLAG anti-body conjugated DynaBeads, which were further released by incubation with elution buffer (refer to S1 Text for details).

#### Tandem mass spectrometry analyses

For Mass spectrometric analyses, samples were run in 1D SDS-PAGE gel and stained by silver nitrate [70]. The SDS-PAGE gels were cut into pieces according to the silver staining result. The gel pieces were digested with trypsin (Promega) overnight, and then analyzed via liquid chromatography combined with electrospray tandem mass spectrometry on an LTQ Orbitrap (Thermo Fisher) with lock mass calibration. All of the raw data files were searched against Mascot Daemon software (Version 2.3.0, Matrix Science, London, UK) based on the Mascot algorithm. The database used was Swiss-Prot (Taxonomy: Arabidopsis Thaliana; release 2012_12_28, with 11571 entries). To reduce false positive identification results, a decoy database containing the reverse sequences was appended. The searching parameters were set up as following: full trypsin (KR) cleavage with two missed cleavage sites was considered. Oxidation on methionine and acetylation of the protein N-terminus were set as variable modifications. The peptide mass tolerance was 20 ppm and the fragment ion tolerance was 1.0 Da. Peptides with Percolater scores exceeding 13 were accepted as correct matches, the FDR is 0.01 (refer to S1 Text for details).

#### Plant materials and growth conditions

*Arabidopsis* mutants *clf-29* (SALK_N521003)[71], *swn-21*[72], *lhp1-6* (SALK_011762)[57], *tfl2-2* (CS3797)[73], *atring1a*,*b*[13] and *atbmi1a*,*b*[12,16] in Col-0 background have been described previously. All plants except *clf-29swn-21* were grown in soil under long day (16 hour) photoperiods at 22°C in green house, and seedlings were harvested after 2 weeks. *clf-29swn-21* was grown on half-strength Murashige and Skoog (MS) medium in the same long day conditions as above, and the whole plants were harvested after 2 weeks. Harvested materials were frozen in liquid nitrogen for total RNA isolation or directly vacuum-infiltrated with formaldehyde crosslinking solution for ChIP assay. Quantitative real-time PCR analysis of ChIPed DNA with specific primers (S8 Table) was performed on the two-color real-time PCR detection system (BIO-RAD, Hercules, CA, USA) using the SYBR Green Realtime PCR Master mix (TOYOBO) to represent the relative methylation levels.

#### H3K27me3 ChIP-seq and RNA sample preparation

Both ChIP-seq and RNA-seq experiments were performed in biological duplicates for *clf-29* and *atbmi1a*,*b*, or in independent lines of *LHP1* mutants *lhp1-6* and *tf*l2-2. Biological replicates of *ring1a*,*b* RNA-seq were performed. ChIP assay was performed with the antibody against H3 trimethyl-Lys 27 (Upstate, USA, Cat. 07–449) as previously described (He et al. 2012). Samples without antibody were used as a negative control. More than 10 ng ChIP DNA or 2 μg total RNA from each sample was used for Illumina library generation following the manufacturer’s instructions (Illumina, http://www.illumina.com/). Library construction and deep sequencing were performed by Genergy Biotechnology Co. Ltd. (Shanghai, China) using Illumina HiSeq 2000 following the manufacturer’s instructions (Illumina). Raw data comprise 50 bp of single-end sequences for ChIP-seq and 100 bp of pair-end sequences for RNA-seq.

#### ChIP-seq, ChIP-chip and RNA-seq data analysis

We started by cleaning the sequencing reads, including removing bases with low quality score (<20) and irregular GC content, cutting sequencing adaptor followed by filtering short reads. As a result, 12–36 million reads with MAPQ>20 were obtained for further analysis (detailed statistics summarized in S2A Table). The cleaned reads were mapped to *Arabidopsis thaliana* genome (TAIR10) using BWA 0.7.5a-r405 [74] for DNA sequencing and TOPHATv2.0.8 [75] for RNA sequencing, both with default settings. Tracks for all sequencing data can be visualized through http://bioinfo.sibs.ac.cn/gb2/gbrowse/tair10/. The genotype for each sample was validated by RNA-seq data (S12 Fig). ChIP-seq results were verified for selected sites by qPCR (S13 Fig).

MACS1.4 [76] was used to identify read enriched regions (peaks). Next, MAnorm, a software designed for quantitative comparison of ChIP-seq datasets [33], was applied to characterize the change of genome-wide H3K27me3 profile in PcG mutants as compared to that of Col-0, including *atbmi1a*,*b*, *atring1a*,*b*, *clf-29* and *tfl2-2*. 3,289 regions with differential binding in at least one of the three comparisons were selected based on the following criteria: P value <1e-3 and |M|>1, where M represents the log2 fold change of binding intensity. K-means clustering was performed to partition these sites according to their M values. The target gene of each peak was defined as the genes closest to a given peak localized around the gene body (from 1 kb upstream of transcription start site (TSS) to transcription end site (TES)), The gene annotation file was downloaded from the TAIR homepage (http://www.arabidopsis.org). Published data sets including FIE ChIP-seq and LHP1 ChIP-chip data were downloaded from Gene Expression Omnibus (GEO) http://www.ncbi.nlm.nih.gov/geo/ with accession numbers GSE48857 [40] and GSE8169 [15], respectively. Peaks for ChIP-seq data were identified using MACS14 [76], and ChIP-chip peaks were identified using TileMap implemented in CisGenome [77]. For IGV viewing [78], we normalized H3K27me3 samples such that the numbers of reads in common peak regions between Col-0 and PcG mutants are the same. For input sample, we fitted Poisson distribution model for read in both input and H3K27me3 peak free regions in Col-0, and made these two Poisson distributions the sample λ value (λ = mean and variance of Poisson distribution).

Permutation test was used to test the significance of peak overlap between LHP1 binding sites and regions with reduced H3K27me3 in *tfl2-2*. Firstly, we shuffled LHP1 binding sites for 1,000 times, and obtained 1,000 random genomic regions with the same length distribution of LHP1 binding sites. Secondly, we calculated and recorded the numbers of these random sites overlapping with LHP1 binding. Since none of the 1000 overlapping numbers exceeds the actual overlap number between LHP1 and regions with reduced H3K27me3 in *tfl2-2*., the P value is thus lower than 1e-3.

Differentially expressed genes were detected by DESeq [79], based on the combined criteria: |log2-foldchange| < 1 and P value <0.05. To dissect the relationship of transcriptomic change across samples, genes with differential expression in at least one of the five mutants (*clf-29*, *lhp1-6*, *tfl2-2*, *atring1a*,*b* and *atbmi1a*,*b*) were collected, resulting in 2,438 genes, which were further classified to 3 groups via k-means clustering. The *clf-29swn-21* double mutant has effect on much larger range of genes, and only the expression change profile for 2,438 genes are shown here. Transcriptomic data for different tissues were downloaded from GENEVESTIGATOR [80]. Gene Set Enrichment Analysis was performed with default settings [43].

The replicates of ChIP-seq and RNA-seq data show good correlation in terms of log_2_ (fold-change) of read intensity between Col-0 and PcG mutants (S14–S16 Figs). Robust Index was calculated for each genomic region or gene to measure the repeatability between replicates. Robust $\text{Index }=\;\frac{\left|\text{log}\left(FC1\right)-\text{log}\left(FC2\right)\right|}{\text{log}\left(FC1\right)+\text{log}\left(FC2\right)}$. FC1 and FC2 represent fold change in replicated dataset 1 and dataset 2, respectively. Genomic regions or genes with lower Robust Index are more credible in terms of the change of binding (i.e. M value) or expression (S3 and S4 Tables).

#### Motif and TF binding enrichment analyses in H3K27me3 peaks

To detect TF binding motifs enriched in H3K27me3 peak regions, we downloaded the position weight matrixes (PWMs) of 269 motifs identified from both mammals and plants collected by JASPAR database [81], and performed motif scan [82] applied to a 1,000 bp window centered at the peak center. For each motif M, the raw motif matching score at each peak P was calculated as: ${\text{max}}_{S\subseteq \;P}\;\left[log\frac{P\left(S|M\right)}{P\left(S|B\right)}\right]$, in which S is a sequence fragment of the same length as the motif, and B is the background frequency of four types of nucleotides (A, C, G, T) estimated from the genome. The enrichment of motif M in a peak list was defined as the ratio of the motif occurrence in the peak list as compared to its occurrence in random genomic regions. Fisher’s exact test was used to calculate the enrichment P value. Motifs enriched with an enrichment P value of 0.01 were presented in a heat map.

To identify TFs whose binding enriched in different peak sets, we collected ChIP-seq data sets from 695 publicly available studies (till May 2015) from Gene Expression Omnibus (GEO http://www.ncbi.nlm.nih.gov/geo/) and processed the data to peak lists as described above. Next, we calculated the enrichment of the overlap between peak set I or peak set II with each of these peak lists using Fisher’s exact test, and the top enriched TF bindings are shown in Fig 6C. The GEO accession numbers, the enrichment statistics, as well as overlapping regions for enriched TFs are listed in S7 Table.

### Data access

The ChIP-seq and RNA-seq data were deposited in Gene Expression Omnibus (GEO http://www.ncbi.nlm.nih.gov/geo/) under the accession number GSE67322. Tracks for all sequencing data and related public data can be visualized through our local genome browser: http://bioinfo.sibs.ac.cn/gb2/gbrowse/tair10/

## Supporting Information

S1 Text Detailed description of experimental procedures for FIE complex purification and tandem mass spectrometry analyses. (PDF)

S1 Fig Distribution of 5,055 Col-0 H3K27me3 peaks in relation to gene annotation. (PDF)

S2 Fig IGV screenshots illustrate regions with quantitative H3K27me3 difference that can’t be characterized by peak overlap analysis. (PDF)

S3 Fig MA plots of all peaks from comparisons of PcG mutants and Col-0 after normalization by MAnorm. (A-D) represent comparisons between Col-0 and (A) *clf-29* (B) *tlf2-2* (C) *atring1a*,*b* and (D) *atbmi1a*,*b*. Each dot represents a peak. X-axis is the A value, which represents the average intensity. Y-axis is the M value, which represents the difference of the intensity. The color range represents -log10 P value associated with normalized peaks. Here, positive M value indicates higher H3K27me3 level in PcG mutants as compared to that in Col-0, and negative M value represents lower H3K27me3 level in PcG mutants. The numbers of regions with elevated or depressed H3K27me3 levels in each mutant as compared to wild type are labeled based on combined criteria |M| > 1 and P value < 1e-3.
(PDF)

S4 Fig Col-0 ChIP-seq read distributions of Col-0 peak regions and peak free regions overlapping with H3K27me3 increased loci in *clf-29* (M>0). (PDF)

S5 Fig Genes specifically induced in *clf-29* as compared to *swn-21* are significantly enriched in gene set in group I repressed by CLF and LHP1 as shown in Fig 4A. (A) Heatmap includes another 1,290 genes whose expressions are only affected in *clf-29swn-21* not depicted in Fig 4A. (B) Enrichment of class 1 genes in group I. ***, Fishers’ exact text P < 1e-3.
(PDF)

S6 Fig Different tissues are partitioned to 8 clusters based on gene expression profile across tissues. K-means clustering is used.
(PDF)

S7 Fig Tissue biased expression analyses of 108 and 164 genes as revealed by GSEA. The microarray expression data were downloaded from GENEVESTIGATOR. Tissues are partitioned to 8 tissue clusters as shown in S6 Fig. GSEA calculated the normalized enrichment score (NES) representing the enrichment of 108 or 164 genes in different tissue biased genes. The x-axis represents all genes targeted by H3K27me3 in Col-0, y axis presents the running enrichment score. Heatmap in Fig 5G summarized the NESs of tissue biased expression of these two gene sets.
(PDF)

S8 Fig The logo and distribution around peak summits for motifs enriched in H3K27me3 peaks identified in Col-0. X-axis represents the distance of given motifs to peak summit (bp), y-axis represent the fraction of motifs located in a given position.
(PDF)

S9 Fig TFs whose bindings associated with peak set I. (A) IGV screenshots showing examples of co-occupancy between H3K27me3 in seedlings and the bindings of MADS-box TFs in inflorescence. The positions of MADS box TF binding motif CArG-box are indicated by pink bars at bottom of the screen shots, and are highlighted by grey area. (B) Those peak set I targets also occupied by floral organ identity TFs show significant inflorescence biased expression.
(PDF)

S10 Fig The phylogenetic tree showing the divergence of CLF and SWN after the origin of angiosperms. (PDF)

S11 Fig IGV screen shots showing examples of co-occupancy between H3K27me3 and SVP in seedlings. (PDF)

S12 Fig IGV screen shots showing the genotype of each mutant as validated by RNA-seq data. (PDF)

S13 Fig ChIP-qPCR validation of ChIP-seq data for selected sites. (A-B) ChIP-qPCR validations of regions with decreased (A) or increased (B) H3K27me3 in *clf-29* and *tfl2-2* as revealed by ChIP-seq data. Top panel is qPCR result. Shown are mean ±s.d. For each loci, input, ChIPed, and negative control samples were repeated for 3 times. Y-axis represents % input = 2^(Ctiput—CtIP)^ -2^(Ctinput-Ctneg)^. CtIP: cycle threshold (Ct) value of samples immunoprecipitated using H3K27me3 antibody; Ctneg: Ct value of negative control, which is the samples immunoprecipitated with beads but without antibody; Ctinput: Ct value of input DNA without immunoprecipitation. Bottom panel shows read count of regions where ChIP-qPCR validations were performed. (C-D) ChIP-qPCR validations of regions with decreased (A) or increased (B) H3K27me3 in *atring1a*,*b* and *atbmi1a*,*b*.
(PDF)

S14 Fig Replicates of ChIP-seq (A) and RNA-seq (B) samples show good correlation in terms of log_2_ fold-change of read intensity. Correlation between M values of ChIP-seq replicates. Left panel: scatter plots in lower left triangle showed the correlation of M values between replicates, and numbers in upper right triangle showed Pearson correlation coefficients, with larger numbers having bigger font sizes; right panel: heatmap showing the correlation coefficients across samples. (A) Boxplots showing high correlation between replicated ChIP-seq data in terms of the M value distribution of peak set I and peak set II shown in Fig 3A. (B) Boxplots showing high correlation between replicated RNA-seq data in terms of the expression change of gene group I and gene group II shown in Fig 4A.
(PDF)

S15 Fig Result in Fig 5C is reproducible using replicated data. (A) Distribution of H3K27me3 changes (measured by M values) for 108 genes and 164 genes in PcG mutants. (B) Distribution of expression changes for 108 genes and 164 genes in PcG mutants.
(PDF)

S16 Fig motifs enriched in regions with reduced H3K27me3 in each mutant. Regions in each PcG mutant with reduced H3K27me3 were identified, followed by motif enrichment analysis. The heatmap shows the enrichment P values in each mutant for motifs shown in Fig 6A.
(PDF)

S1 Table FIE co-purifies with PcG proteins. (XLSX)

S2 Table Statistics of ChIP-seq and RNA-seq reads, and Col-0 H3K27me3 peaks, targets, and enriched biological functions and motifs. (A) ChIP-seq and RNA-seq read statistics for each sample. (B) 5,055 H3K27me3 peaks identified in Col-0, as well as list of peak target, which is defined as the nearest gene whose gene body is located within 1 kb upstream or 1 kb downstream of the peak region. (C) Biological functions enriched in Col-0 H3K27me3 peak targets, including GO terms of biological process (BP), molecular function (MF), cellular compartment (CC), and Interpro domains. (D) Motifs enriched in 5,055 Col-0 H3K27me3 peaks with P<1e-5, as well as the enrichment statistics of CArG box motifs.
(XLSX)

S3 Table Statistics of differentially H3K27me3-marked regions identified based on MAnorm. (A) Genomic coordinates, motifs and target genes information for 3,289 H3K27me3 regions regulated by at least one PcG component with cutoff: |M|>1 & P<1e-3. (B) 51% regions with reduced H3K27me3 in clf-29 show less reduction in summit regions. (C) 53% regions with reduced H3K27me3 in tfl2-2 show less reduction in summit regions.
(XLSX)

S4 Table List of differentially expressed genes in PcG mutants, expression class, as well as the enriched functions for different classes of genes. (A) 2,438 differentially expressed genes in PcG mutants with the following criteria: |FC|>1 and P <0.05 in at least one mutant. (B) Biological functions enriched in Col-0 H3K27me3 peak targets, including GO terms, Interpro domains, and SP_PIR keywords. (C) Biological functions enriched in Col-0 H3K27me3 peak targets, including GO terms, Interpro domains, and SP_PIR keywords.
(XLSX)

S5 Table Lists of target genes whose expression and H3K27me3 are controlled by different combinations of PcGs. (A) 108 target genes whose expression and H3K27me3 are controlled by CLF and LHP1. (B) 164 target genes whose expression and H3K27me3 are controlled by RING1 and BMI1.
(XLSX)

S6 Table List of PcG targets with inflorescence (A) or embryo (B) biased expression. (A) 108 genes ranked by their extent of inflorescence biased expression. Column B is the enrichment score of GSEA, and Column C represents whether the given gene has strong inflorescence biased expression as returned by leading edge analysis implemented in GSEA. (NOTE: low expressed genes across RNA-seq samples (read density in given gene <2) were removed before GSEA). (B) 164 genes ranked by their extent of embryo biased expression. Column B is the enrichment score of GSEA, and Column C represents whether the given gene has strong embryo biased expression as returned by leading edge analysis implemented in GSEA. (NOTE: low expressed genes across RNA-seq samples (read density in given gene <2) were removed before GSEA).
(XLSX)

S7 Table TFs whose bindings are enriched in peak set I. The enrichment statistics and co-occupied genomic regions are listed.
(XLSX)

S8 Table Primers used in this study. (XLSX)
