# Correction: Arabidopsis Flower and Embryo Developmental Genes are Repressed in Seedlings by Different Combinations of Polycomb Group Proteins in Association with Distinct Sets of Cis-regulatory Elements

**DOI:** 10.1371/journal.pgen.1006574

**Published:** 2017-01-25

**Authors:** Hua Wang, Chunmei Liu, Jingfei Cheng, Jian Liu, Lei Zhang, Chongsheng He, Wen-Hui Shen, Hong Jin, Lin Xu, Yijing Zhang

There are two errors in S1 Text under the subheading “Affinity purification”. In the sixth sentence of this paragraph, "2.5 hours with 200 ml of Dynabeads" should read "2.5 hours with 200μL of Dynabeads". In the last sentence of this paragraph, "incubation with 500 ml of elution buffer" should read "incubation with 500μL of elution buffer". Please view the correct S1 Text below.

## Supporting Information

S1 Text Detailed description of experimental procedures for FIE complex purification and tandem mass spectrometry analyses. (DOCX)
