# Supplementary material for: Arabidopsis Flower and Embryo Developmental Genes are Repressed in Seedlings by Different Combinations of Polycomb Group Proteins in Association with Distinct Sets of Cis-regulatory Elements
Source: PLoS Genet. 2016 Jan 13;12(1):e1005771. doi: 10.1371/journal.pgen.1005771 (PMC4711971; doi:10.1371/journal.pgen.1005771)
Supplement: S1 Text — (PDF) [file pgen.1005771.s001.pdf]

SUPPLEMENTAL METHODS FOR

Arabidopsis Flower and Embryo Developmental Genes are Repressed in Seedlings by Different Combinations of Polycomb Group Proteins in Association with Distinct Sets of Cis-regulatory Elements

This file contains the detailed description of experimental procedures for FIE complex purification and tandem mass spectrometry analyses.

Construction of pFIE: FLAG-FIE plants

To construct pFIE: FLAG-FIE plants, we used 2.5kb upstream of start codon as promoter, and 1.3kb downstream of stop codon as terminator. Promoter, genebody and terminator sequences of *FIE* were cloned into pCAMBIA1300 and transformed into *fie-2*+/-(GK-534F01-020364) by floral dip with *Agrobacterium tumefaciens* (strain GV 3101). FLAG tag was introduced with FIE-pro-R primer. After two generations, transgenic plants were validated by PCR with primers including FIE-Flat-TDNA-F, FIE-Flat-TDNA-R, o8409 and FIE-TDNA-F were used (primers listed in Supplemental Table 7). Plants with negative PCR result using FIE-Flat-TDNA-F and FIE-Flat-TDNA-R and positive PCR result using o8409 and FIE-TDNA-F were verified as pFIE:FLAG-FIE/*fie-/-* (pFIE:FLAG-FIE) lines, which were used for subsequent experiment.

Affinity purification

Approximately 15 g of leaf explants (8 days cultured in callus induced medium) from pFIE: FLAG-FIE transgenic T2 plants, or from Col-0 plants as a negative control, were ground in liquid nitrogen, and the powder was divided into 6 parts. Each part was resuspended in 50 mL of ChIP lysis buffer (CLB: 50mM HEPES pH7.5, 150mM NaCl, 1mM EDTA, 1% TritonX-100,10% glycerol, 0.35%β-Mercaptoethanol and 1 protease inhibitor cocktail tablet). The tissue was then rotated for 30 min at 4 ºC and centrifuged at 4ºC for 20 minutes at 4000 rpm. The supernatant was discarded and the precipitate was resuspended in 2ml lysis buffer (LB: 50 mM Tris pH7.6, 150 mM NaCL, 5 mM MgCl2, 10% glycerol, 0.1% NP-40, 0.5 mM DTT, 1 mg/mL pepstatin and 1 protease inhibitor cocktail

tablet). The tissue was then rotated for 30 min at 4ºC and centrifuged at 4ºC for 20 minutes at 132000 rpm (centrifuged for another 10min if needed). Each supernatant was incubated at 4ºC for 2.5 hours with 200 mL of Dynabeads conjugated with FLAG anti-body conjugated DynaBeads, (Cristea and Chait 2011). The FLAG beads were then washed 2 times with 10 mL of LB and four times with 1 mL of LB. For each wash, the beads were rotated at 4ºC for 5 minutes. Proteins were then released from the FLAG beads by incubation with 500 mL of elution buffer(EB: （0.5N）NH_4OH, （0.5mM）EDTA), and then concentrated to 20μl at 4ºC.

Protein Digestion

For Mass spectrometric analyses, samples were run in 1D SDS-PAGE gel and stained by silver nitrate {Mortz, 2001 #52}. The SDS-PAGE gels were cut into pieces according to the silver staining result. The band pieces were washed with 300 ul of dH2O water for 5 min then discard the supernatant. Add 100 ul of destaining solution (100mM potassium ferricyanide; 50mM sodium thiosulfate) for 10min. Discard the supernatant. Wash the band pieces with 400 ul of 50 mM NH4HCO3 / CH3CN (50:50 v/v) for 10 min. Discard the supernatant. Add 100 ul of CH3CN to dehydrate the band pieces for 10 min. Discard the supernatant. Add 20 ul of 10 ng/μl of modified trypsin in 20 mM NH4HCO3. Allow bands to rehydrate in trypsin digestion buffer for 30 min and then add more 20 mM NH4HCO3 (minus the trypsin) to cover the band pieces. Incubate at 37℃overnight. Add an equal volume of CH3CN to the digest. Incubate at room temperature for 30 min. Transfer supernatant to a new clean Eppendorf tube and then lyophilized by vacuum centrifugation and stored at -80℃

Liquid Chromatography-Mass Spectrometry

Liquid chromatography was performed on a nano Acquity UPLC system (Waters Corporation, Milford, USA) connected to a LTQ Orbitrap XL mass spectrometer (Thermo Scientific, Bremen, Germany) equipped with an online nano-electrospray ion source (Michrom Bioresources, Auburn, USA). Peptides were resuspended with 25 μl solvent A (5% acetonitrile, 0.1% formic acid in water). 20μl peptide solution was loaded onto the Captrap Peptide column (2mm x 0.5mm, Michrom Bioresources, Auburn, USA) at a 20 μl/min flow rate of solvent A for 5 min and then was separated on a Magic C18AQ reverse phase

column (100μm id×15cm, Michrom Bioresources, Auburn, USA) with a linear gradient. Starting from 5% B (90% acetonitrile, 0.1% formic acid in water) to 45% B (in other words, from 95% A to 55% A, the same below) in 70 min. The column flow rate was maintained at 500 nL/min and column temperature was maintained at 35℃. The electrospray voltage of 1.4 kV versus the inlet of the mass spectrometer was used.

LTQ Orbitrap XL mass spectrometer was operated in the data-dependent mode to switch automatically between MS and MS/MS acquisition. Survey full-scan MS spectra with one microscan (m/z 350-1600) was acquired in the Obitrap with a mass resolution of 60,000 at m/z 400, followed by MS/MS of the eight most-intense peptide ions in the LTQ analyzer. The automatic gain control (AGC) was set to 1000 000 ions, with maximum accumulation times of 500 ms. For MS/MS, we used an isolation window of 2 m/z and the automatic gain control (AGC) of LTQ was set to 20 000 ions, with maximum accumulation time of 120 ms. Single charge state was rejected and dynamic exclusion was used with two microscans in 10 s and 90 s exclusion duration. For MS/MS, precursor ions were activated using 35% normalized collision energy at the default activation q of 0.25 and an activation time of 30 ms. The spectrum were recorded with Xcalibur (version 2.0.7) software.

Data Processing and Analysis

The mass spectra were searched using the Mascot Daemon software (Version 2.3.0, Matrix Science, London, UK) based on the Mascot algorithm. The database used to search was the UniProtKB/Swiss-Prot database (Taxonomy: Arabidopsis Thaliana; release 2012_12_28, with 11571 entries). To reduce false positive identification results, a decoy database containing the reverse sequences was appended to the database. The searching parameters were set up as follows: full trypsin (KR) cleavage with two missed cleavage was considered. Oxidation on methionine and aceytlation of the protein N-terminus were set as variable modifications. The peptide mass tolerance was 20 ppm and the fragment ion tolerance was 1.0 Da. Peptides which Percolater scores exceeding13 were accepted as correct matches, the FDR is 0.01.
