# Supplementary figures and images for: Arabidopsis Flower and Embryo Developmental Genes are Repressed in Seedlings by Different Combinations of Polycomb Group Proteins in Association with Distinct Sets of Cis-regulatory Elements
Source: PLoS Genet. 2016 Jan 13;12(1):e1005771. doi: 10.1371/journal.pgen.1005771 (PMC4711971; doi:10.1371/journal.pgen.1005771)

**S1 Fig**

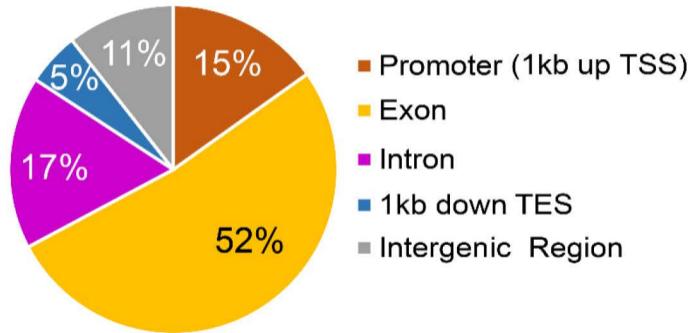

Supplement: S1 Fig — (PDF) [file pgen.1005771.s002.pdf]

# S3 Fig

A

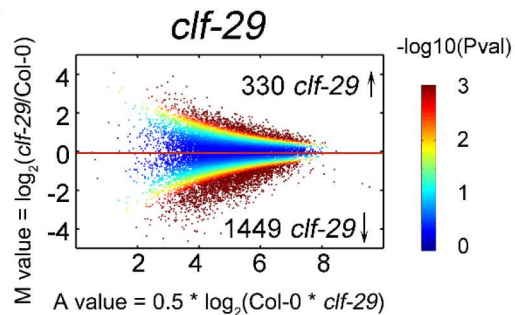

B

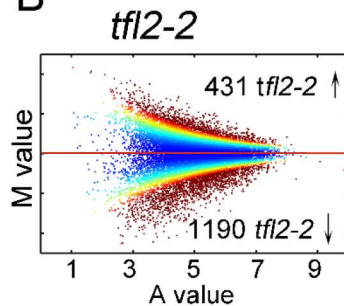

C

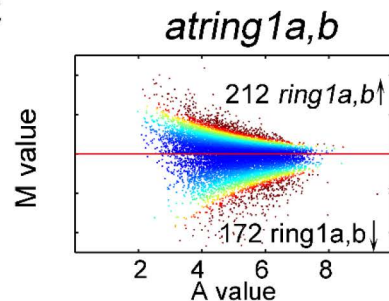

D

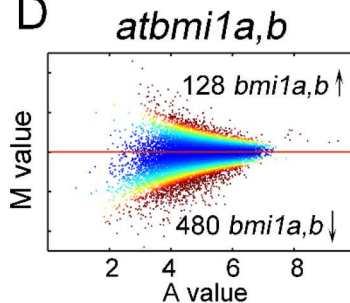

Supplement: S3 Fig — (A-D) represent comparisons between Col-0 and (A) clf-29 (B) tlf2-2 (C) atring1a,b and (D) atbmi1a,b. Each dot represents a peak. X-axis is the A value, which represents the average intensity. Y-axis is the M value, which represents the difference of the intensity. The color range represents -log10 P value associated with normalized peaks. Here, positive M value indicates higher H3K27me3 level in PcG mutants as compared to that in Col-0, and negative M value represents lower H3K27me3 level in PcG mutants. The numbers of regions with elevated or depressed H3K27me3 levels in each mutant as compared to wild type are labeled based on combined criteria |M| > 1 and P value < 1e-3. (PDF) [file pgen.1005771.s004.pdf]

# S4 Fig

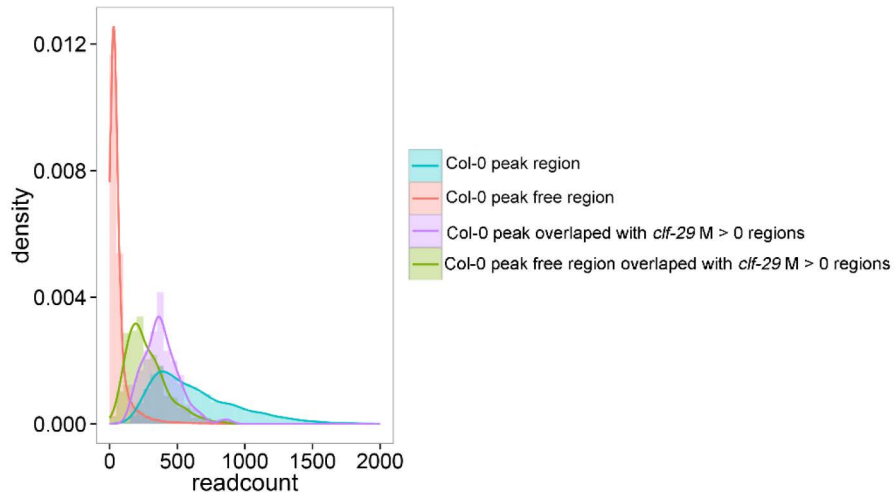

Supplement: S4 Fig — (PDF) [file pgen.1005771.s005.pdf]

# S5 Fig

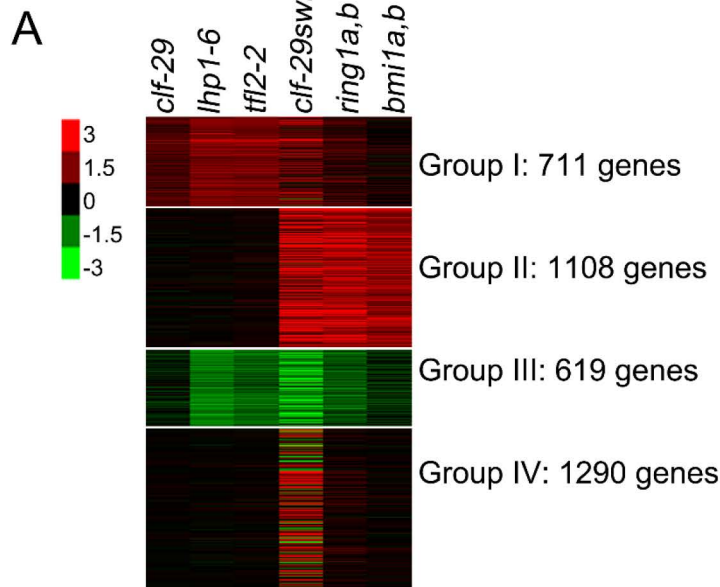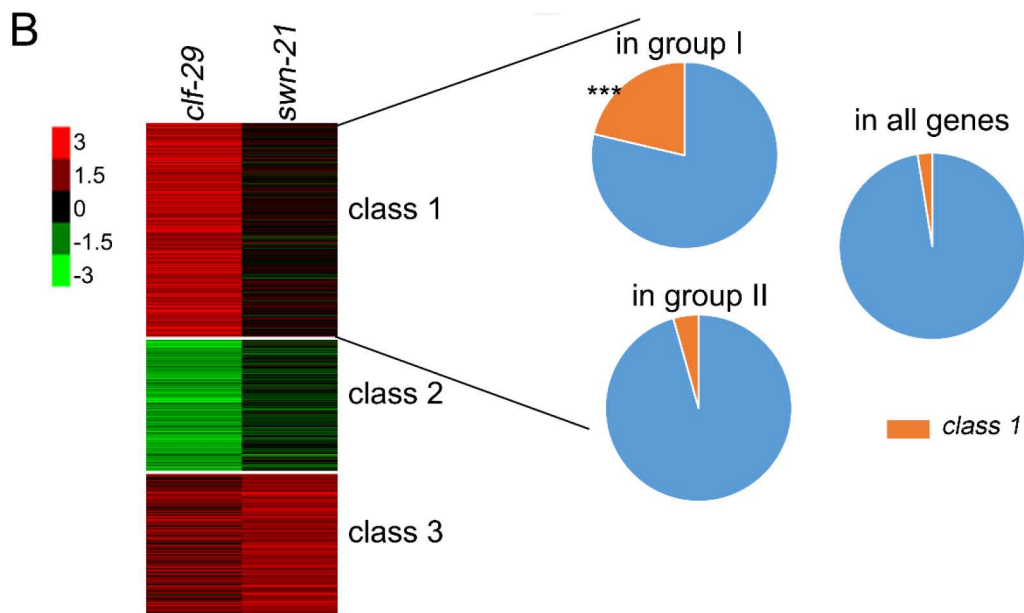

Supplement: S5 Fig — (A) Heatmap includes another 1,290 genes whose expressions are only affected in clf-29swn-21 not depicted in Fig 4A. (B) Enrichment of class 1 genes in group I. ***, Fishers’ exact text P < 1e-3. (PDF) [file pgen.1005771.s006.pdf]

# S6 Fig

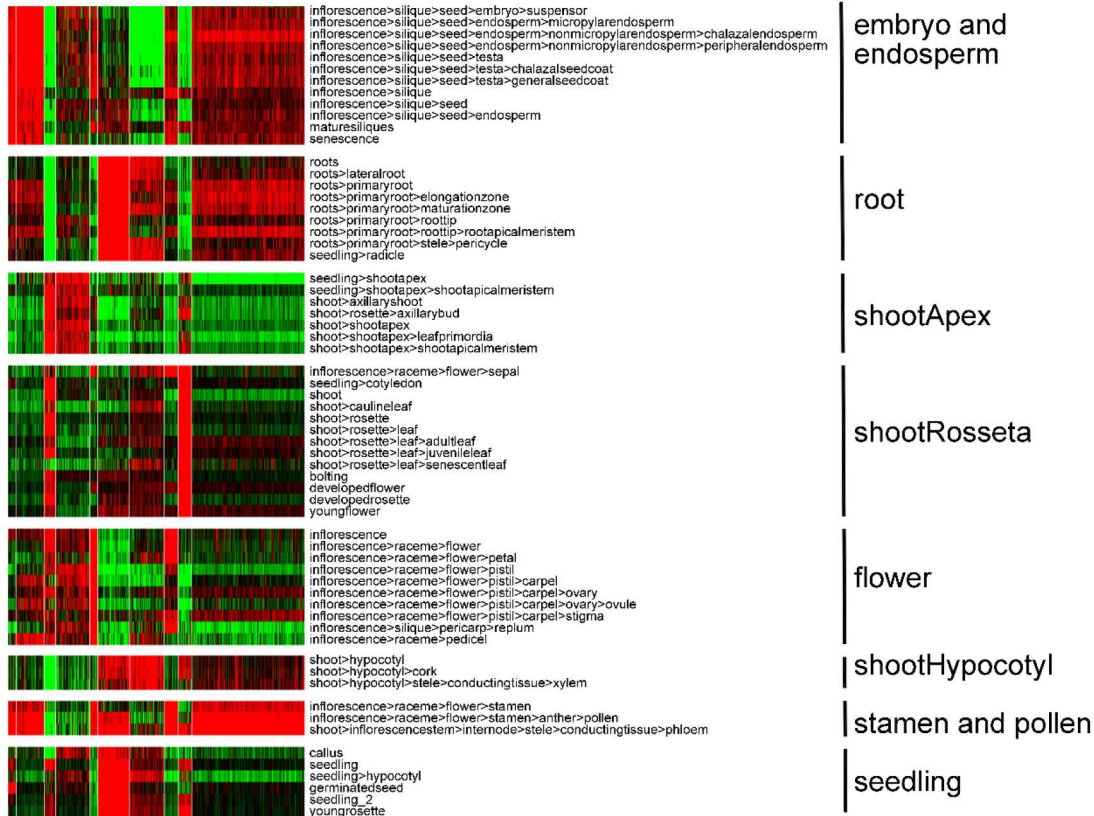

Supplement: S6 Fig — K-means clustering is used. (PDF) [file pgen.1005771.s007.pdf]

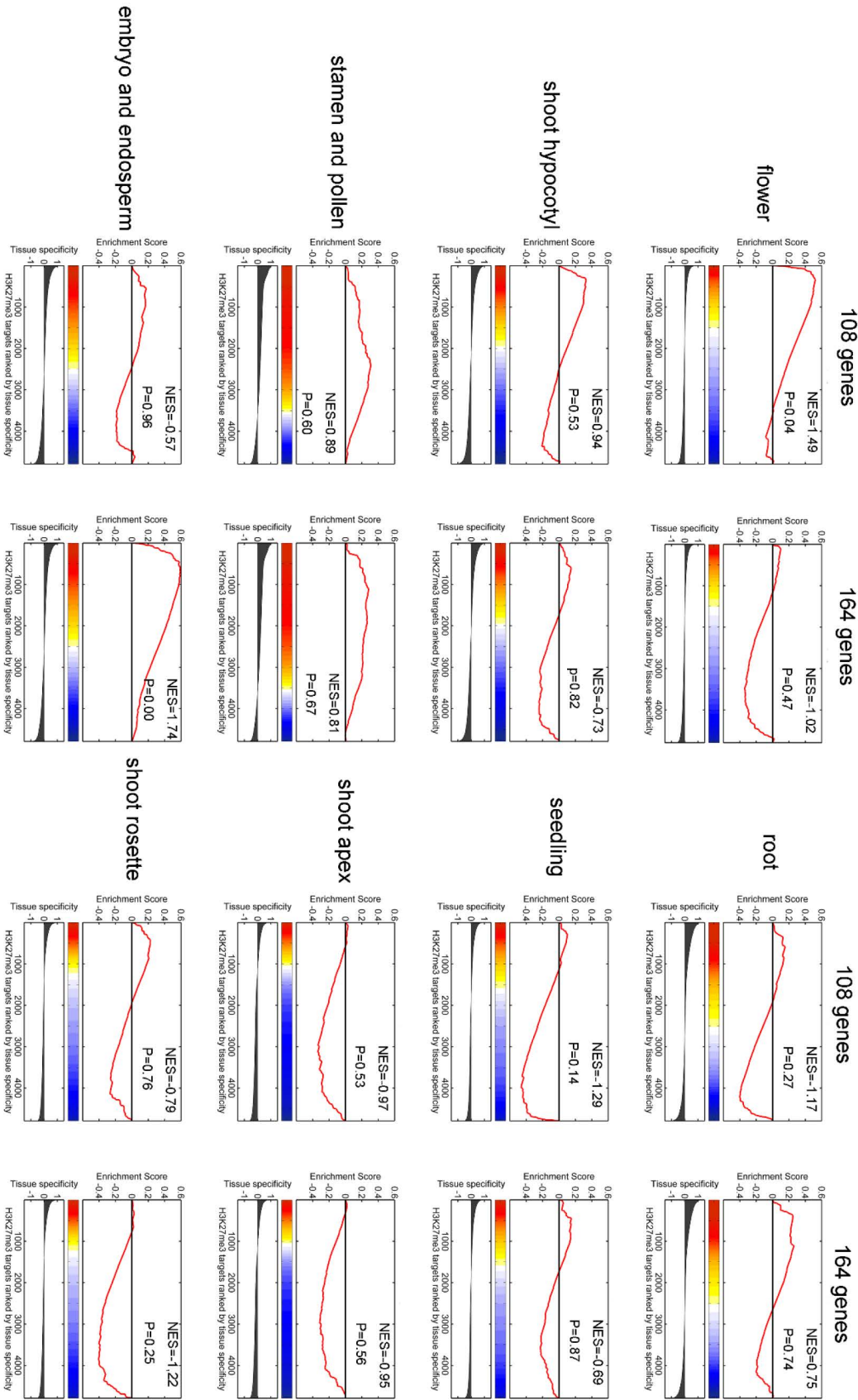

Supplement: S7 Fig — The microarray expression data were downloaded from GENEVESTIGATOR. Tissues are partitioned to 8 tissue clusters as shown in S6 Fig. GSEA calculated the normalized enrichment score (NES) representing the enrichment of 108 or 164 genes in different tissue biased genes. The x-axis represents all genes targeted by H3K27me3 in Col-0, y axis presents the running enrichment score. Heatmap in Fig 5G summarized the NESs of tissue biased expression of these two gene sets. (PDF) [file pgen.1005771.s008.pdf]

# S8 Fig

LEC2/ABI3/FUS3

FC = 2.23

P = 2.85E-79

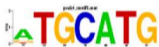

SMZ

FC = 1.38

P = 3.99E-18

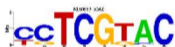

AtMYB84

FC = 1.40

P = 4.18E-14

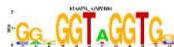

ABF1

FC = 1.33

P = 2.85E-13

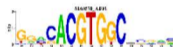

ABI4

FC = 1.30

P = 2.60E-11

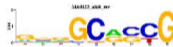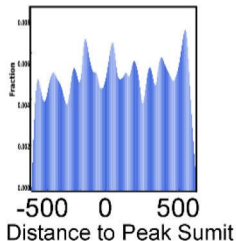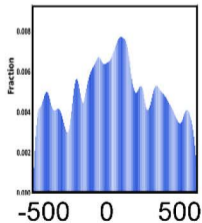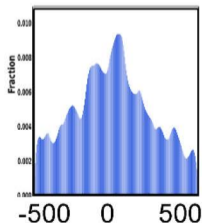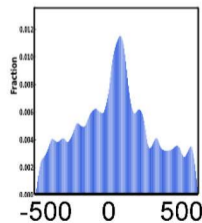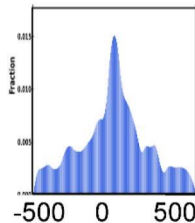

Supplement: S8 Fig — X-axis represents the distance of given motifs to peak summit (bp), y-axis represent the fraction of motifs located in a given position. (PDF) [file pgen.1005771.s009.pdf]

# S11 Fig

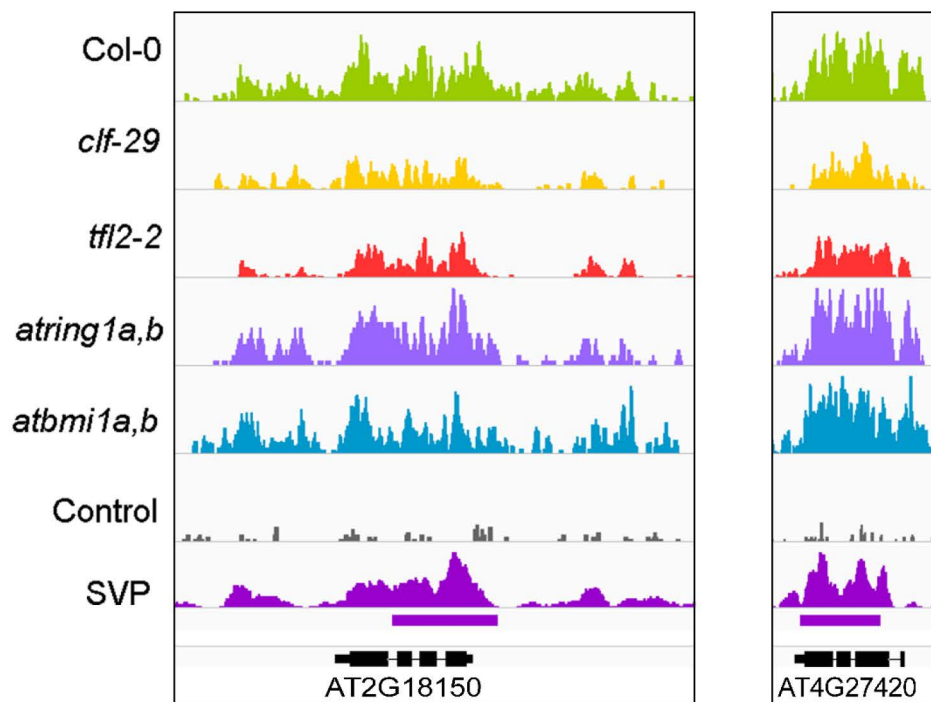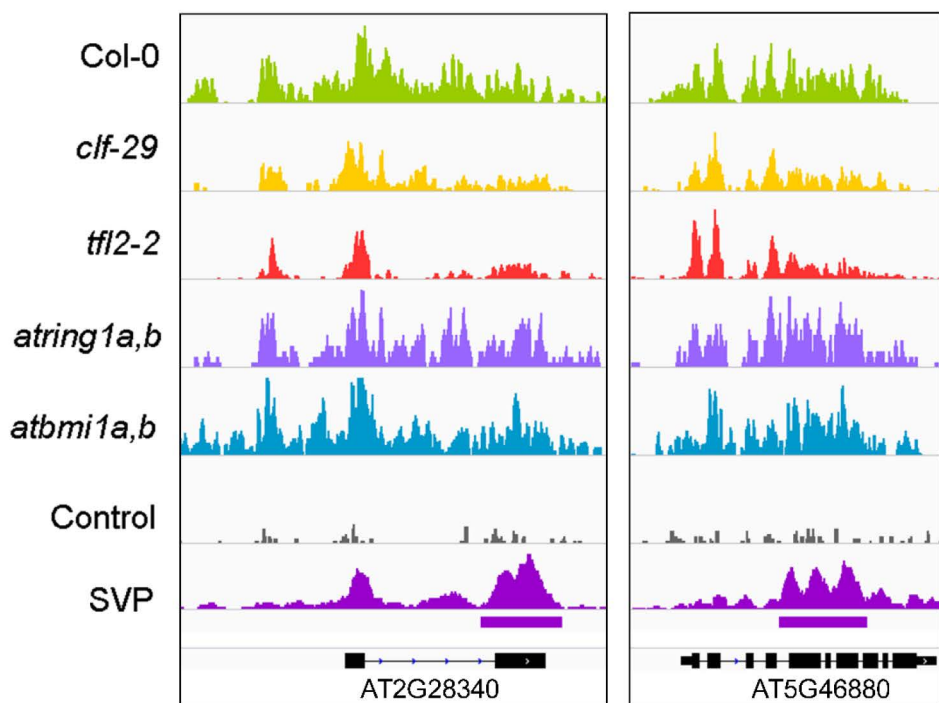

Supplement: S11 Fig — (PDF) [file pgen.1005771.s012.pdf]

# S13 Fig

Col-0

*clf-29*

*tfl2-2*

*atring1a,b*

*atbmi1a,b*

A

ChIP-qPCR

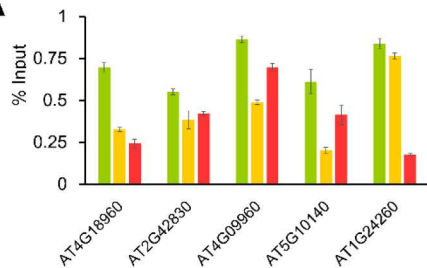

ChIP-seq

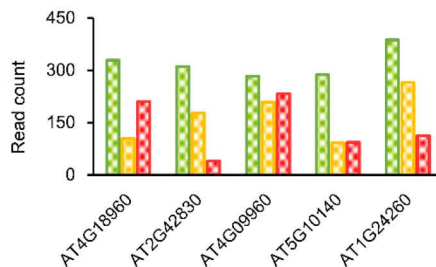

B

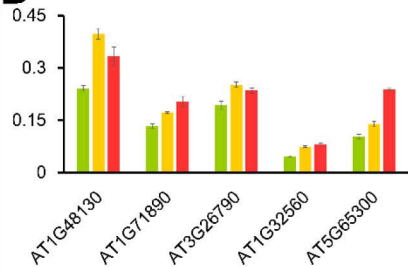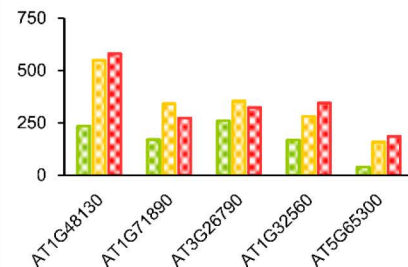

C

ChIP-qPCR

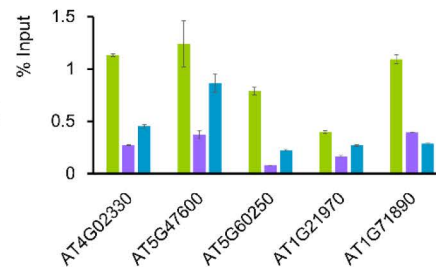

ChIP-seq

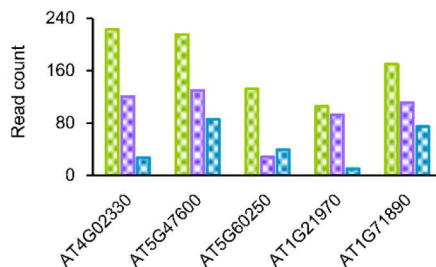

D

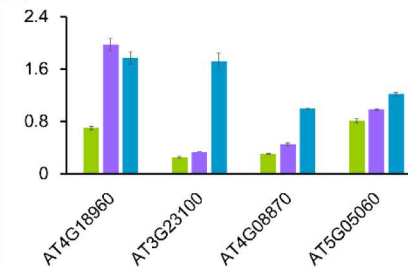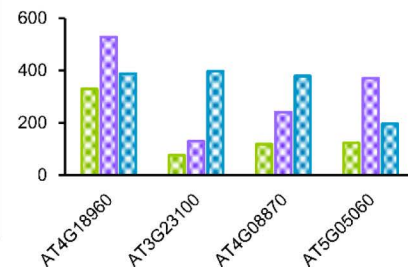

Supplement: S13 Fig — (A-B) ChIP-qPCR validations of regions with decreased (A) or increased (B) H3K27me3 in clf-29 and tfl2-2 as revealed by ChIP-seq data. Top panel is qPCR result. Shown are mean ±s.d. For each loci, input, ChIPed, and negative control samples were repeated for 3 times. Y-axis represents % input = 2(Ctiput—CtIP) -2(Ctinput-Ctneg). CtIP: cycle threshold (Ct) value of samples immunoprecipitated using H3K27me3 antibody; Ctneg: Ct value of negative control, which is the samples immunoprecipitated with beads but without antibody; Ctinput: Ct value of input DNA without immunoprecipitation. Bottom panel shows read count of regions where ChIP-qPCR validations were performed. (C-D) ChIP-qPCR validations of regions with decreased (A) or increased (B) H3K27me3 in atring1a,b and atbmi1a,b. (PDF) [file pgen.1005771.s014.pdf]

# S14 Fig

A

## Person correlation of M values between replicates

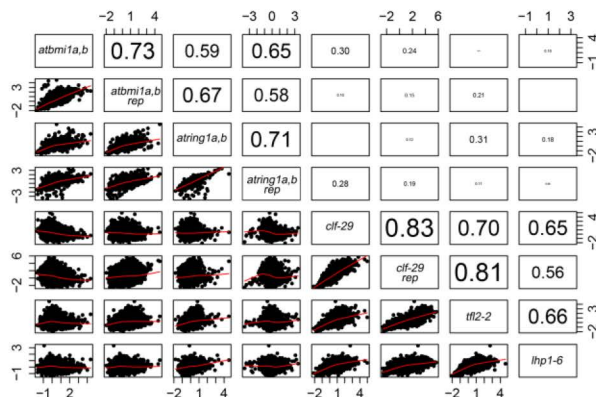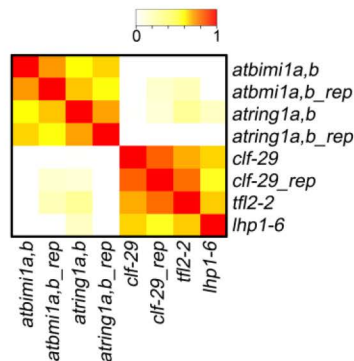

B

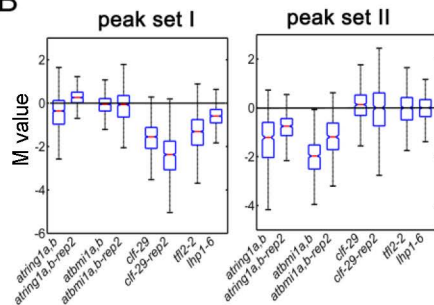

C

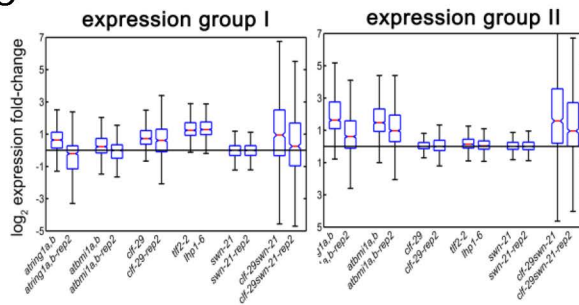

Supplement: S14 Fig — Correlation between M values of ChIP-seq replicates. Left panel: scatter plots in lower left triangle showed the correlation of M values between replicates, and numbers in upper right triangle showed Pearson correlation coefficients, with larger numbers having bigger font sizes; right panel: heatmap showing the correlation coefficients across samples. (A) Boxplots showing high correlation between replicated ChIP-seq data in terms of the M value distribution of peak set I and peak set II shown in Fig 3A. (B) Boxplots showing high correlation between replicated RNA-seq data in terms of the expression change of gene group I and gene group II shown in Fig 4A. (PDF) [file pgen.1005771.s015.pdf]

# S15 Fig

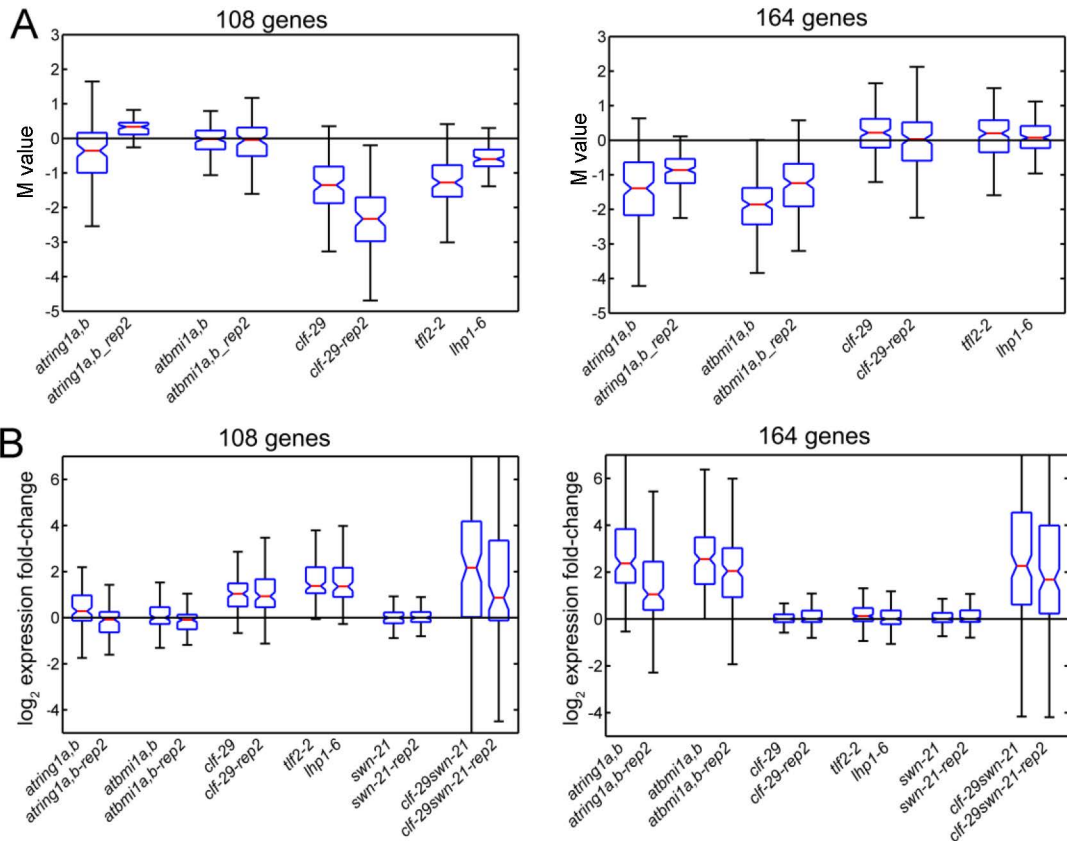

Supplement: S15 Fig — (A) Distribution of H3K27me3 changes (measured by M values) for 108 genes and 164 genes in PcG mutants. (B) Distribution of expression changes for 108 genes and 164 genes in PcG mutants. (PDF) [file pgen.1005771.s016.pdf]

S16 Fig

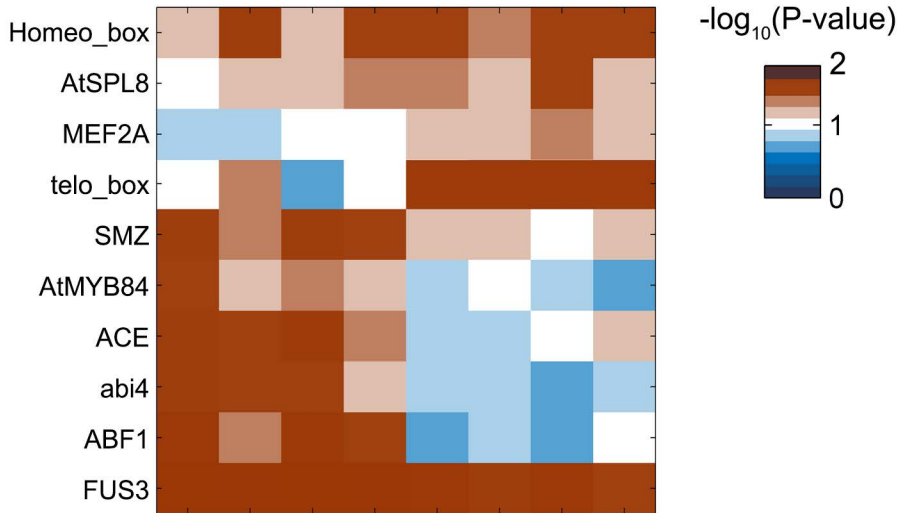

Supplement: S16 Fig — Regions in each PcG mutant with reduced H3K27me3 were identified, followed by motif enrichment analysis. The heatmap shows the enrichment P values in each mutant for motifs shown in Fig 6A. (PDF) [file pgen.1005771.s017.pdf]
