# Supplementary material for: Arabidopsis Flower and Embryo Developmental Genes are Repressed in Seedlings by Different Combinations of Polycomb Group Proteins in Association with Distinct Sets of Cis-regulatory Elements
Source: PLoS Genet. 2016 Jan 13;12(1):e1005771. doi: 10.1371/journal.pgen.1005771 (PMC4711971; doi:10.1371/journal.pgen.1005771)
Supplement: S2 Fig — (PDF) [file pgen.1005771.s003.pdf]

S2 Fig

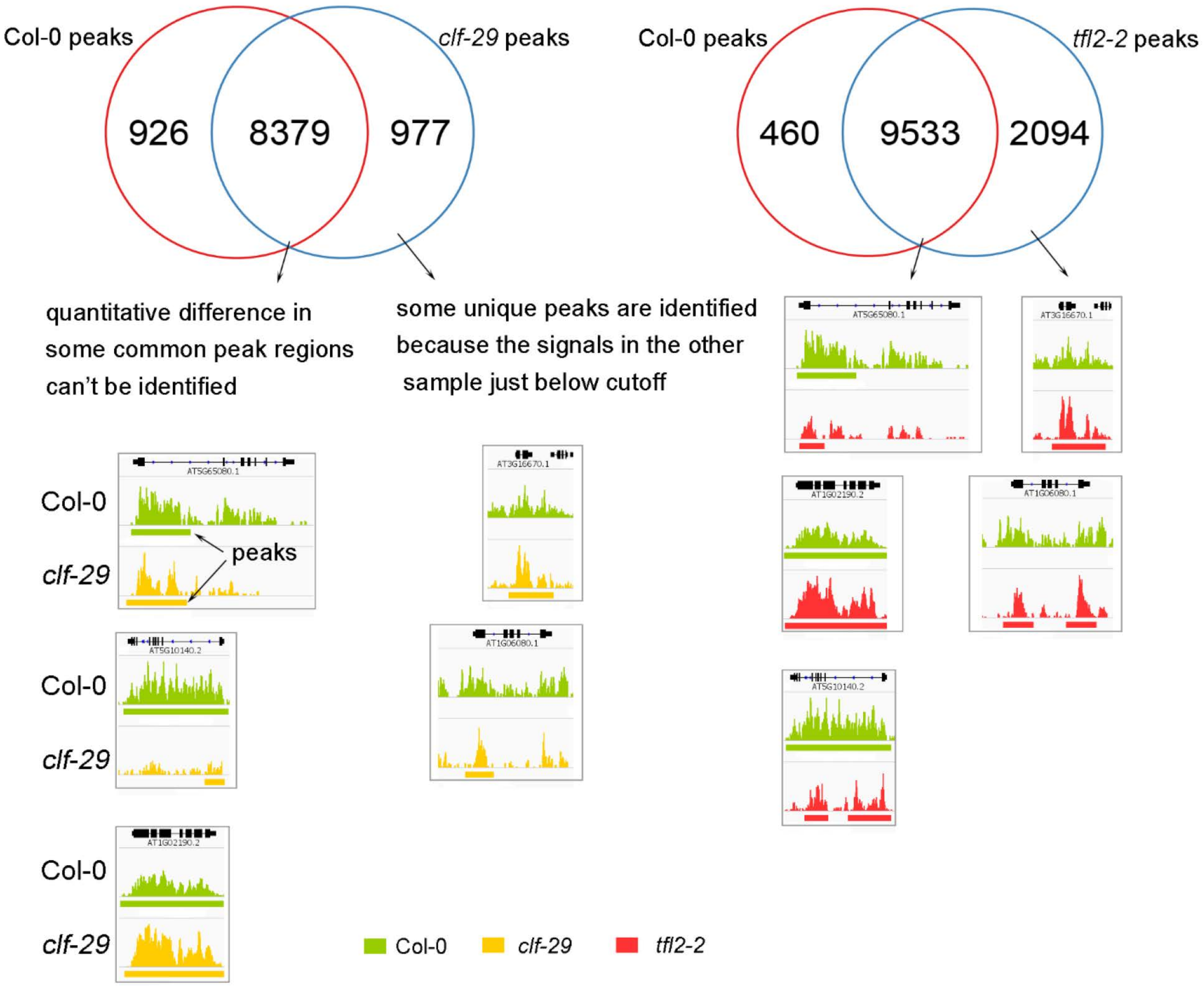

Col-0 peaks
clf-29 peaks
926
8379
977
Col-0 peaks
tfl2-2 peaks
460
9533
2094
quantitative difference in some common peak regions can't be identified
some unique peaks are identified because the signals in the other sample just below cutoff
AT5G65080.1
AT3G16670.1
AT1G02190.2
AT1G06080.1
AT5G10140.2
Col-0
clf-29
peaks
Col-0
clf-29
Col-0
clf-29
Col-0
clf-29
tfl2-2
