# Supplementary material for: Arabidopsis Flower and Embryo Developmental Genes are Repressed in Seedlings by Different Combinations of Polycomb Group Proteins in Association with Distinct Sets of Cis-regulatory Elements
Source: PLoS Genet. 2016 Jan 13;12(1):e1005771. doi: 10.1371/journal.pgen.1005771 (PMC4711971; doi:10.1371/journal.pgen.1005771)

S9 Fig

A

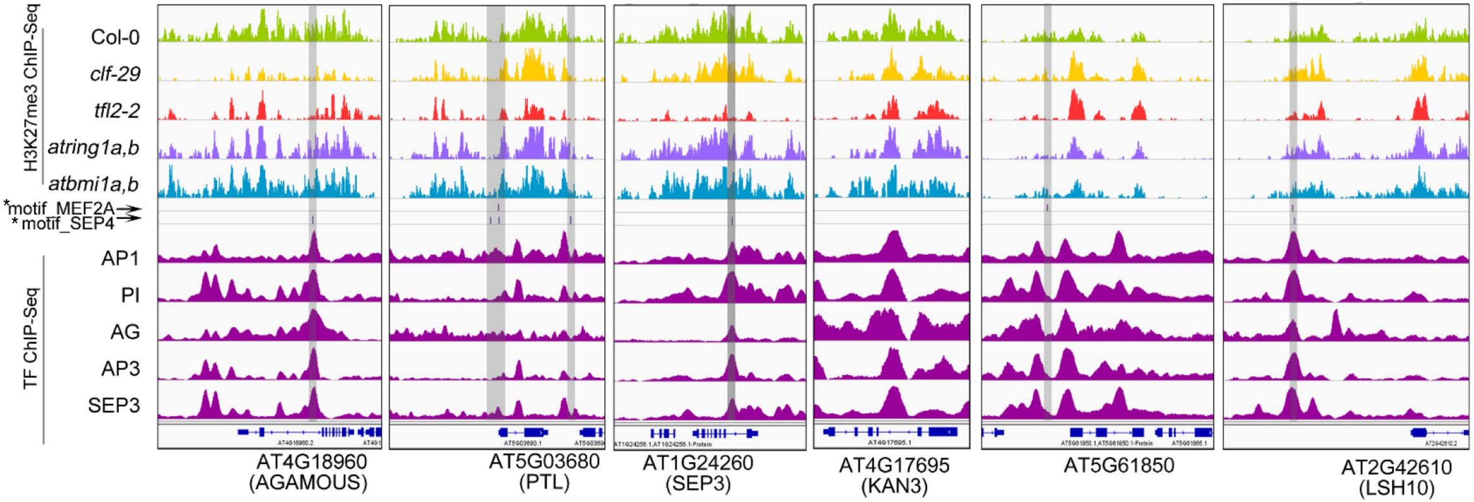

* MEF2A and SEP4 are representatives of CArG-box motifs, whose motif logos could be found in Table S3A.

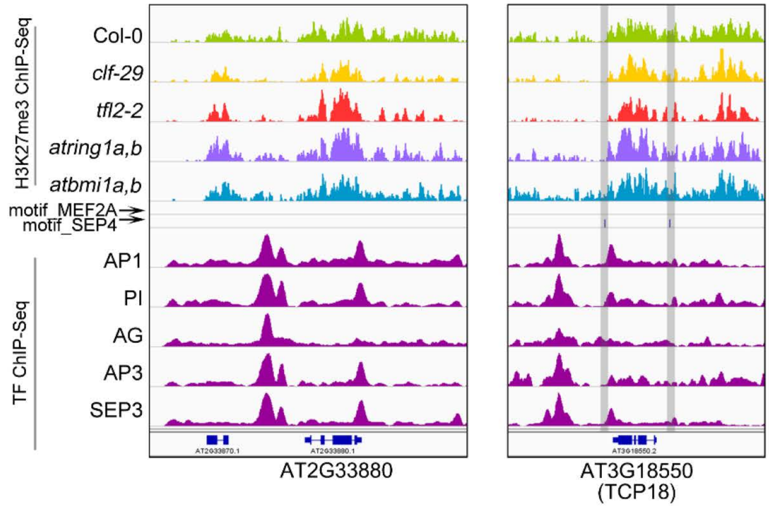

B

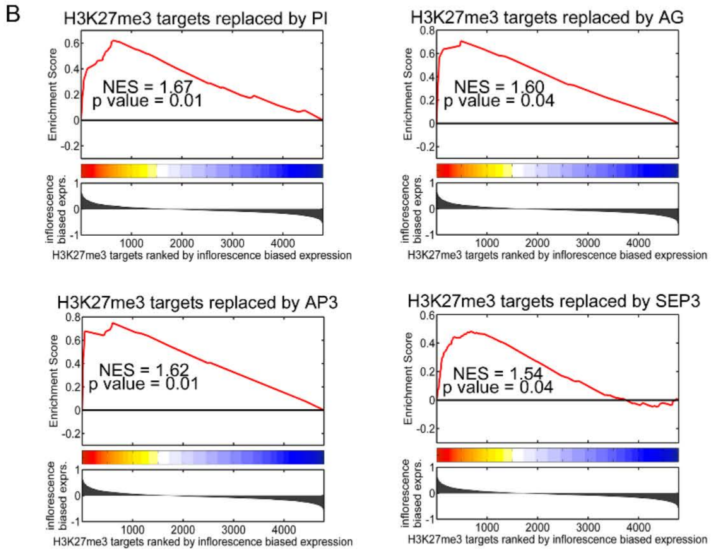

Supplement: S9 Fig — (A) IGV screenshots showing examples of co-occupancy between H3K27me3 in seedlings and the bindings of MADS-box TFs in inflorescence. The positions of MADS box TF binding motif CArG-box are indicated by pink bars at bottom of the screen shots, and are highlighted by grey area. (B) Those peak set I targets also occupied by floral organ identity TFs show significant inflorescence biased expression. (PDF) [file pgen.1005771.s010.pdf]
