# Supplementary material for: Arabidopsis Flower and Embryo Developmental Genes are Repressed in Seedlings by Different Combinations of Polycomb Group Proteins in Association with Distinct Sets of Cis-regulatory Elements
Source: PLoS Genet. 2016 Jan 13;12(1):e1005771. doi: 10.1371/journal.pgen.1005771 (PMC4711971; doi:10.1371/journal.pgen.1005771)
Supplement: S10 Fig — (PDF) [file pgen.1005771.s011.pdf]

S10 Fig

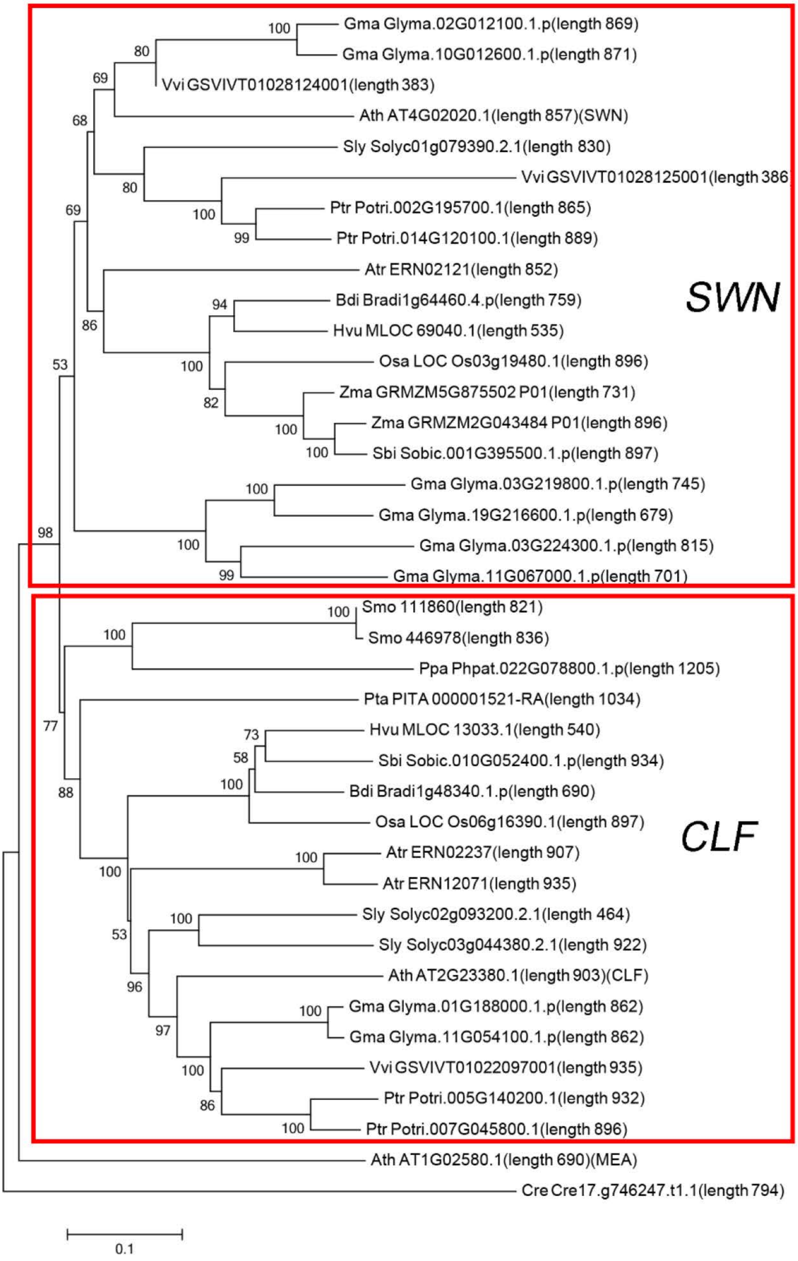

Abbreviaions

Ath: *Arabidopsis thaliana*

Bdi: *Brachypodium distachyon*

Cre: *Chlamydomonas reinhardtii*

Atr: *Amborella trichopoda*

Gma: *Glycine max*

Zma: *Zea mays*

Vvi: *Vitis vinifera*

Osa: *Oryza sativa*

Hvu: *Hordeum vulgare*

Ppa: *Physcomitrella patens*

Pta: *Pinus taeda*

Ptr: *Populus trichocarpa*

Sbi: *Sorghum bicolor*

Sly: *Solanum lycopersicum*

Smo: *Selaginella moellendorffii*
