# Supplementary material for: Arabidopsis Flower and Embryo Developmental Genes are Repressed in Seedlings by Different Combinations of Polycomb Group Proteins in Association with Distinct Sets of Cis-regulatory Elements
Source: PLoS Genet. 2016 Jan 13;12(1):e1005771. doi: 10.1371/journal.pgen.1005771 (PMC4711971; doi:10.1371/journal.pgen.1005771)
Supplement: S12 Fig — (PDF) [file pgen.1005771.s013.pdf]

S12 Fig

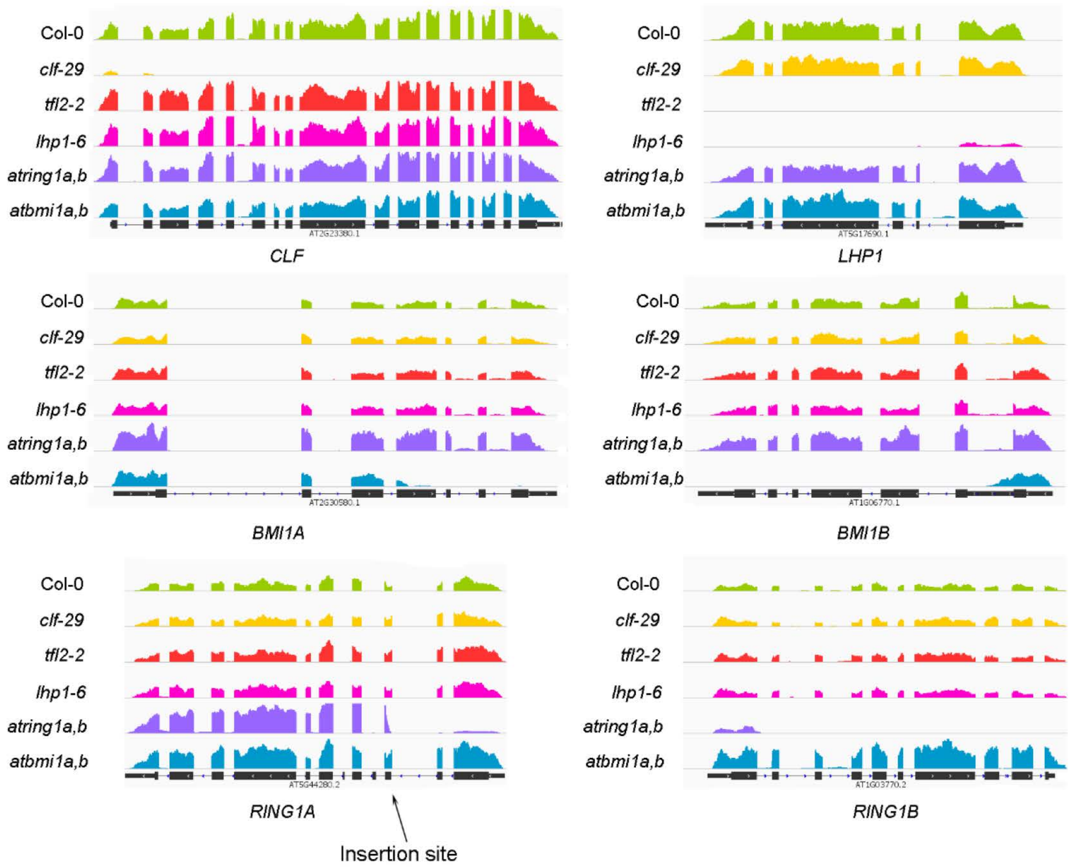

almost no expression of1st and 2nd exons

Expression of other exons are driven by T-DNA insertion,which has ATG on 5' end

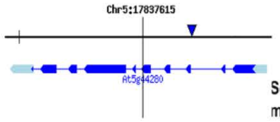

Sequence (A. th genome BLAST matches underlined)

```
>25-K015443-022-293-A04-8409
TTTACGCTCTGAAAAAAATGCAGACACAATTAAACAAGAAAGATCCACTCCAGCTTCTTT
ACTTCTACAGTAAAGAATCTACTCTCAAGACAAAAATAAAGCTGACACTCTAAAGGTCAA
GTTAAATAGAAGATGAATCACTTCTTAGGACACAACTTCCAGTAATGGCCGCTATTACTT
CAAGGTAAAGACTTTACACTTGTTTAGCCACAAGAGCGTAGAGGTTTTGAGCAGTAATAC
GAGAGGAATTACAACATGCAAGTGCTAAGAAGATGCATCAACAGATTACTAAATCAAGCA
GAGCAATTTTAACATTTAATTGACTATGGGATCGTCATAANNGGGTAGTGGGAATCTAAT
ATGACTTGTTGGCATGTGTCCTGATTGATTATAACAACTTACACCGCAGCTCGATCTTTA
TAAGCCTA
```

GenBank Accession AL945948 [GenBank]

Graphic View
